# On a high photocatalytic activity of high-noble alloys Au–Ag/TiO_2_ catalysts during oxygen evolution reaction of water oxidation

**DOI:** 10.1038/s41598-022-06608-7

**Published:** 2022-02-16

**Authors:** Anum Shahid Malik, Taifeng Liu, Meena Rittiruam, Tinnakorn Saelee, Juarez L. F. Da Silva, Supareak Praserthdam, Piyasan Praserthdam

**Affiliations:** 1 High-Performance Computing Unit (CECC-HCU), Center of Excellence on Catalysis and Catalytic Reaction Engineering (CECC), Chulalongkorn University, Bangkok, 10330 Thailand; 2 Center of Excellence on Catalysis and Catalytic Reaction Engineering (CECC), Chulalongkorn University, Bangkok, 10330 Thailand; 3 National & Local Joint Engineering Research Center for Applied Technology of Hybrid Nanomaterials, Henan University, Kaifeng, 475004 China; 4 Rittiruam Research Group, Bangkok, 10330 Thailand; 5 Saelee Research Group, Bangkok, 10330 Thailand; 6 São Carlos Institute of Chemistry, University of São Paulo, PO Box 780, São Carlos, SP 13560-970 Brazil

**Keywords:** Chemical engineering, Heterogeneous catalysis, Photocatalysis, Density functional theory, Chemical physics, Chemical physics

## Abstract

The analysis via density functional theory was employed to understand high photocatalytic activity found on the Au–Ag high-noble alloys catalysts supported on rutile TiO_2_ during the oxygen evolution of water oxidation reaction (OER). It was indicated that the most thermodynamically stable location of the Au–Ag bimetal-support interface is the bridging row oxygen vacancy site. On the active region of the Au–Ag catalyst, the Au site is the most active for OER catalyzing the reaction with an overpotential of 0.60 V. Whereas the photocatalytic activity of other active sites follows the trend of Au > Ag > Ti. This finding evident from the projected density of states revealed the formation of the trap state that reduces the band gap of the catalyst promoting activity. In addition, the Bader charge analysis revealed the electron relocation from Ag to Au to be the reason behind the activity of the bimetallic that exceeds its monometallic counterparts.

## Introduction

In the current era, solar energy is confined for practical reasons and has increased the attention of both academia and industrial sectors. The essential worldwide goal of solar energy is to create electricity, and it is prospective to generate fuels from the waste of CO_2_ and H_2_O by induction of solar photochemistry. In the past 40 years, heterogeneous photocatalysis and photochemistry fields have developed comprehensively in return to demanding energy and environmental issues^1–8^. One of the most capable solutions for the energy problem is photocatalytic overall water oxidation (POWS), i.e., oxygen and hydrogen evolution^9,10^. Water electrolysis—one of the most practical routes to produce hydrogen to establish clean and renewable energy cycles, accounted for up to 4% of the global hydrogen production^11,12^. Water electrolysis entails two half-cell reactions: the cathodic hydrogen evolution (HER) and anodic oxygen evolution (OER) reactions.

The metal/semiconductor photocatalysts have vast POWS potential and have drawn broad attention for a long time^13^. Since 1972, a typical semiconductor TiO_2_ exhibited a suitable band structure for POWS as a photocatalyst^14^. Moreover, the TiO_2_-supported Pt catalysts are competent of POWS under the range of ultraviolet light^15^. Evidently, the visible light POWS shows a higher potential for practical applications. However, due to the large bandgap of TiO_2_, TiO_2_-based photocatalysts POWS driven by visible light remains a great challenge^16–18^. Even though the bandgap of TiO_2_ could be effectively narrowed down by elemental doping to harvest visible light, the energy levels introduced by impurity dopants can act as recombination centers for electrons and holes, resulting in a poor photocatalytic activity^19,20^.

Moreover, it has been reported that various metals, e.g., Pt, Au, Ag, Pd, Ru, Cu, etc., loaded on semiconductors materials, e.g., TiO_2_, RuO_2_, etc. to confine photoexcited electrons through the formed Schottky junction can result in reserved charge recombination for photocatalytic enhancement^21–23^. When the material is operated in a visible light range, Au, Ag, and Cu can act as metallic plasmons and trigger localized surface plasmon resonance (LSPR)^24,25^. This energizes the electrons above the Fermi level from the occupied energy levels^26,27^. These electrons with high energy pass through the Schottky barrier of metal–semiconductor can be located in the semiconductor conduction band (CB), contributing to the competent charge separation and the photocatalytic reduction in the visible light region^28,29^. In the meantime, the positive charges left behind in the separated energy bands of plasmonic metals can be used to drive various oxidation reactions^30^.

Therefore, metal-loaded TiO_2_ (M/TiO_2_, M = Au, Ag, Cu, etc.) plasmonic photocatalysts can be good candidates for the POWS that work under visible light. The Au, Ag, Cu plasmonic metals act as electron donors and generate electrons into TiO_2_ under visible light. The differences in electron transfer from TiO_2_ to the metal or metal to TiO_2_ could drive different routes of water redox reaction resulting in various products distribution^31–33^. Silver (Ag) has been regarded as a promising candidate over gold (Au) due to its high catalytic activity. Water oxidation is a sluggish reaction in nature and artificial photosynthetic systems. Nevertheless, the plasmon resonance frequency of Ag is in the near-ultraviolet region, limiting its photocatalysis applications in the visible light region^34^. Consequently, the application of Ag-based photocatalysts remains challenging^35^.

A substitute method to stabilize the Ag-based photocatalyst is to alloy the Ag metal with stable metals such as Au. These alloys comprising high content of noble metals are regarded as the high-noble alloys. In dentistry, such materials exhibit increased resistance to the corrosive environment–high stability^36^. When Ag is alloyed with Au, the stability is still maintained. The alloy could produce optical regulation due to their different plasmon response range and produce a prospect to tune the electronic structure, affecting Schottky barrier height and the plasmon-induced charges potentials^37^. In heterogeneous catalysis, although bimetallic alloys have been investigated extensively^38–40^, the reliance of photocatalytic oxygen evolution reactions (OER) on the alloy components for water oxidation reaction is yet to be studied. The DFT calculations can be coupled with the experimental techniques to understand the enhanced activity of heterogeneous catalysts, e.g., Pd–Cu alloy nanoparticles supported on carbon support^41^ and M_4_/CeO_2_ (ZrO_2_) where M_4_ is Pt, Pd, and Rh^42^. In addition, there are other theoretical and experimental works that have been carried out on similar interfaces. For instance, TiO_2_-nanocluster adsorption on Ag and Au noble metal surfaces compared with that on graphene surfaces was recently investigated using density functional theory calculation^43^. This demonstrated that the electronic properties of the cluster on Ag and Au are not different, which helps to understand the experimental results^44^. The bimetallic nanocluster on a finite-size TiO_2_ nano-wire was designed and presented as electronic properties, which are motivating to explore that in other bimetallic groups^45^. The importance of TiO_2_ nanostructure and its energy application in terms of an experimental realization is summarized^46^.

In this work, using DFT calculations and computational hydrogen electrode (CHE), we have evaluated the catalytic performance of Au–Ag/TiO_2_ high noble alloys catalysts during water oxidation together with the investigation on roles of Au in such bimetallic cluster via the Au–Ag bimetallic cluster supported on rutile TiO_2_ (110) models.

## Results and discussion

### Electronic properties of the catalysts: density of states analyses

**Figure 1:**
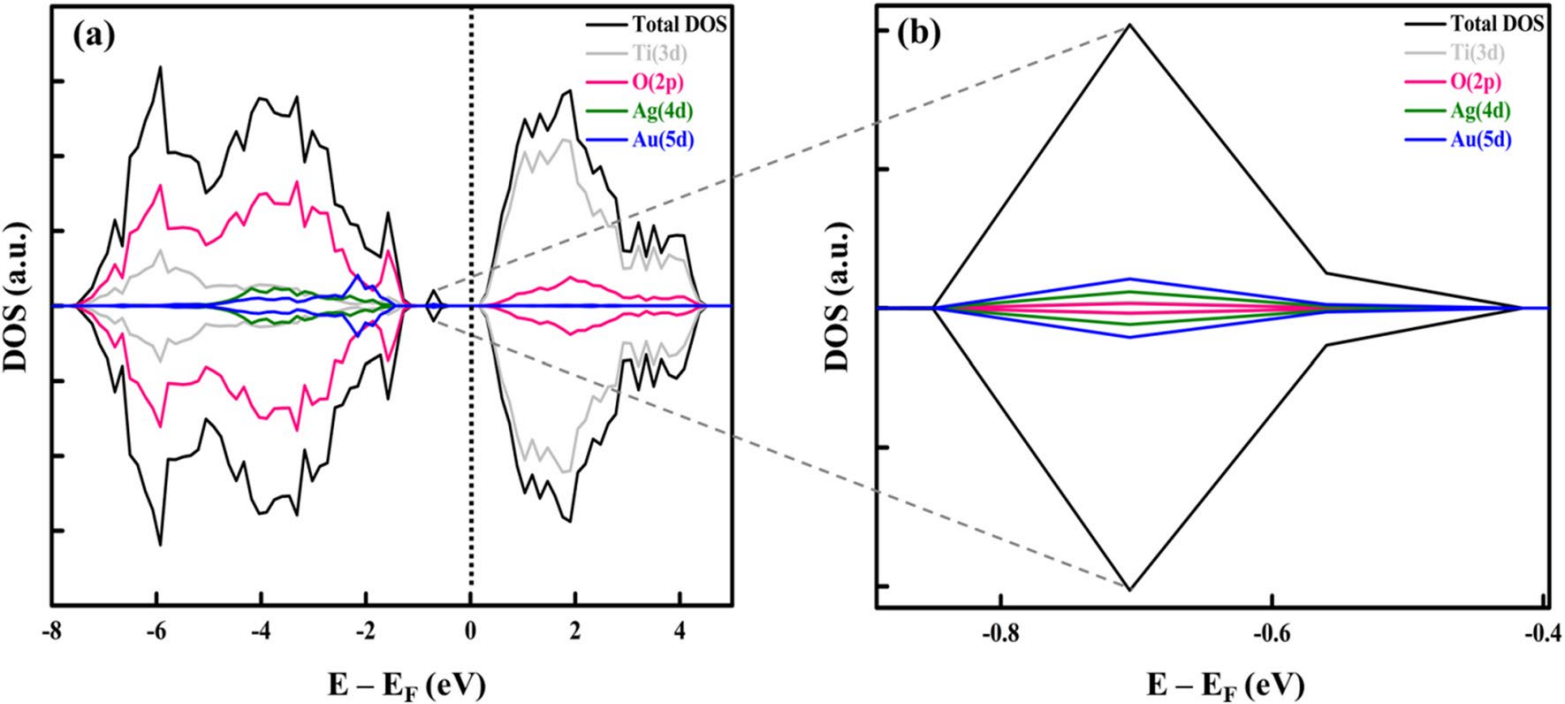
The density of states (DOS) profiles of (**a**) Au_2_–Ag_2_/TiO_2_ (four atoms) cluster on bridging row oxygen vacant site and (**b**) the enlarged view of trap states which is majorly composed of Au(5d) and Ag(4d). (The DOS profile reflects the activity of the catalyst based on the analysis of bonding and antibonding states located at the left side of the E_F_ and right side of the E_F_ regions, respectively, where they are separated by the Fermi level designated at E_F_ = 0 eV.

We employed the HSE06 hybrid functional in DFT calculations for studying the band structure of the most stable structure, i.e., Au_2_–Ag_2_/TiO_2_ embedded in place of oxygen vacancy. We used the Au_2_–Ag_2_ tetrahedral cluster supported on the rutile (110) surface due to its high stability^47^. The density of states (DOS) profiles for Au_2_–Ag_2_/TiO_2_ are shown in Fig. 1. The valence band comprises O (2p), while the conduction band is majorly contributed by Ti (3d). In the forbidden gap, there are hybrid gap states found composed of Au (5d) and Ag (4d), with a significant contribution from Au (5d). The Ag-5 s orbital is much higher in energy than the Au-6 s orbital energy, so a partial charge transfer from Au to Ag occurs^48^.

Consequently, Au atoms tend to be negatively charged, while Ag atoms tend to be positively charged. The partial charge transfer from Au atoms to Ag atoms gives remarkable electrostatic stabilization, making the alloy formation more favorable than pure gold and silver clusters. Therefore, the equivalent mixing between Au and Ag atoms in the alloy formation will likely be more preferential. For Au_4_/TiO_2_ and Ag_4_/TiO_2_ tetrahedral structures positioned on bridging oxygen rows, vacancy results are shown in S.I (see fig S10 and S11).

It is well known that gold clusters have the lowest spin multiplicity as the ground state^49^. Previously, the spin effects of Au clusters on TiO_2_ (110)^50^ and other metal oxides^51^ were studied. As a result, spin and electron distributions were assumed to have no effects in the case of noble metal atoms. Figure 1 shows that the spin-up and spin-down states of the DOS profiles of Ag 4d and Au 5d in Ag_4_Au_4_/TiO_2_ are symmetrical, while in the case of Au_4_/TiO_2_, it exhibits slightly asymmetrical DOS shown in Figure S10 of the supplementary document.

TiO_2_ surface can donate electrons and is ascribed to excess electrons from the defect state found inside the bandgap^34,52–54^. Ag and Au, relatively electronegative atoms, prefer to take the missing oxygen when creating an oxygen vacancy. Pulling an oxygen atom out of the bridging oxygen atom releases electron density, creating a vacancy site. This excess electron is transferred to Ag and Au atoms binding to the vacancy site. This implies that the support plays an active role in catalytic activity because it alters its chemical properties by transferring charge to Ag and Au^55^. In this work, DFT analyses are employed to understand the water oxidation mechanism on the interface between Au/Ag high noble alloys catalysts supported on rutile TiO_2_ (110) and such a mechanism on the support itself. Thus, two main issues are focused, (1) determination of active regions on the surface (2) thermodynamically stable size and location of the active site cluster of initial (Au/TiO_2_, Ag/TiO_2_, Au–Ag/TiO_2_) and cluster models (Au_4_/TiO_2_, Ag_4_/TiO_2_, Au_2_–Ag_2_/TiO_2_).

### Catalytic performance of TiO_2_ support during oxygen evolution reaction (OER)

**Figure 2:**
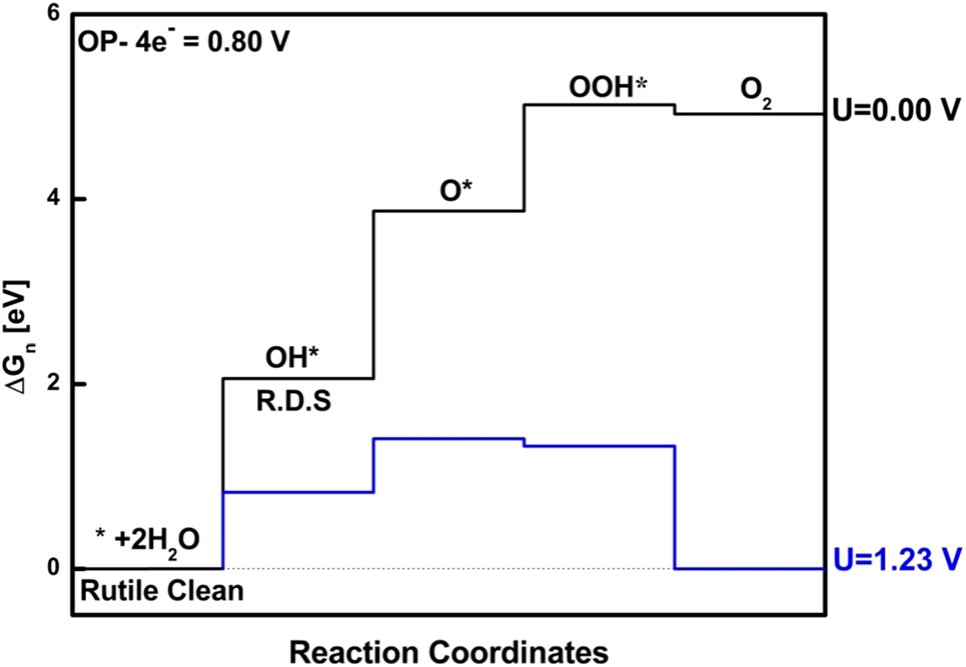
Free energy diagrams for OER through peroxo species on TiO_2_ rutile (110) under external potential U = 0.00 V. (Black profile = O_2_ evolution pathways go via the peroxo O* intermediate. The rate-determining steps (R.D.S) are indicated with an arrow. The overpotential is displayed in the upper left corner. (recreated from^33^).

We first discuss the water oxidation mechanism on clean rutile (110) TiO_2_ surface. In Fig. 7d, we showed the pure surface of TiO_2_ rutile (110), which is well investigated by many researchers theoretically and experimentally. The reported overpotential value for the pure surface is 0.80 eV with the rate-determining step OH* as shown with recreation in Fig. 2^33,56^. Furthermore, Malik et al. and Norskov et al. reported that on the clean or pure surface of TiO_2_ mechanism of water oxidation proceeds through surface-bound peroxo O* species. Thus, Ti was the only metal active site choice for studying water oxidation mechanisms.

### Determination of active regions on the catalyst

Now we started analyzing with initial models of Au/TiO_2_, Ag/TiO_2_, and Au–Ag/TiO_2_, where the model shown in Fig. 1a comprises one Ag atom replacing the bridging oxygen atom on the TiO_2_ surface, while in Fig. 7b, one Au atom substituted an oxygen atom on the TiO_2_ surface. When we studied Au–Ag/TiO_2_ model (Fig. 7c), we tried different positioning of Au and Ag atoms, such as placing both atoms next to each other in the same bridging row or placing them on the same bridging row with the gap of one bridging oxygen atom in between Au and Ag (In S.I. see figure S1). The most stable position was when Au and Ag atoms were positioned on two bridging oxygen vacant sites in different rows, as shown in Fig. 7c. To study the OER mechanism on the Au–Ag/TiO_2_ slab structure, the Ti atom was first chosen as an active site, and then the calculations were performed by choosing Au and Ag as an active site. For Au/TiO_2_ and Ag/TiO_2_ slab structures, the free energy diagrams are shown in S.I. in Figure S8 (a) and (b). The Ti active site was selected far from noble metal atoms. The OER data of the initial model for Fig. 7c, i.e., Au–Ag/TiO_2_, is listed in Table 1. The OER data for Fig. 7a,b are shown in S.I. in table S2, and intermediates are shown in Figure S3 and S4.

**Table 1:** The relative free energies for each type of active site on the bimetallic Au–Ag/TiO_2_ system shown in Fig. 7c, starting from the dissociation of a water molecule (H_2_O + * ↔ H* + HO*) set as the reference point with zero energy.

Active site	ΔG_OH*_	ΔG_O*_	ΔG_OOH*_	Overpotential (V)	Rate-determining steps
Au	1.54	3.88*	4.84	1.11	O* + H_2_O → HOO* + ½H_2_
Ag	0.68	3.29*	4.98	1.38	O* + H_2_O → HOO* + ½H_2_
Ti	0.03	0.17	3.58*	2.18	HOO* → O_2_ + ½H_2_

The propagating step is denoted as ΔG_OH*_, ΔG_O*_, and ΔG_OOH*_ shown together with their overpotentials and rate-determining step. *The rate-determining steps.

**Figure 3:**
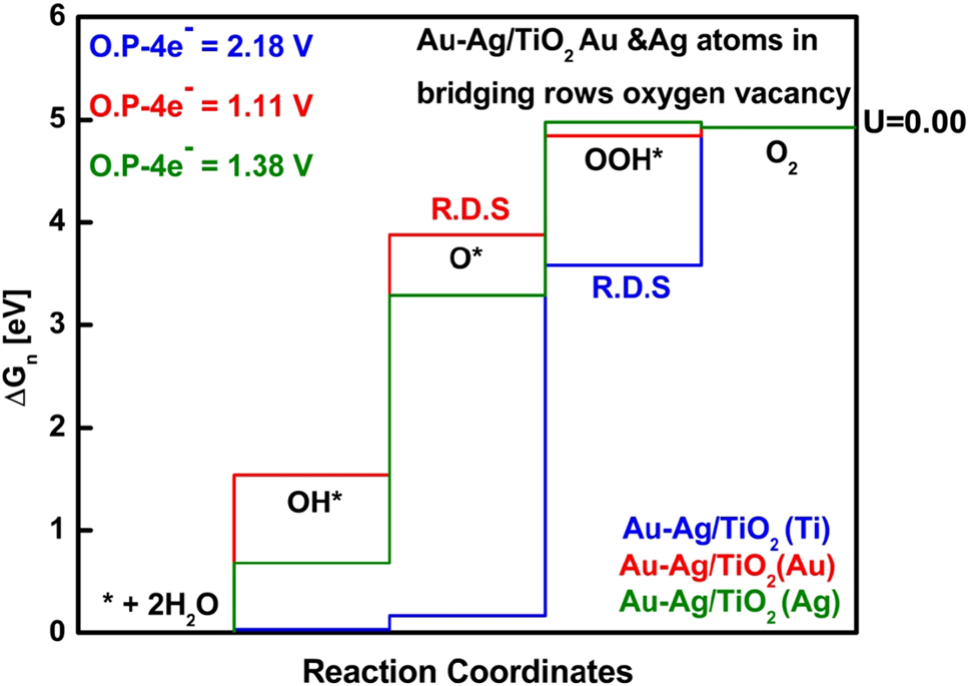
Comparative free energy diagram for Au–Ag/TiO_2_ (Au and Ag placed in bridging vacant oxygen site) slab structure. Blue, red, and green profiles represent Ti, Au, and Ag as active sites. Overpotential for all energy profiles are shown in the upper left corner. The rate-determining step for Au and Au as active sites is O* while OOH* for Ti atom as an active site. R.D.S is rate-determining step.

From the OER results shown above in Fig. 3, it is indicated that the Au atom is preferably the active site with the lowest overpotential with an initial model. However, since Au and Ag atoms are placed on different bridging rows of oxygen vacancy sites, there is no interaction between noble metal atoms on the TiO_2_ surface.

### OER performance of TiO_2_ supported Au–Ag high-noble alloys catalysts

An initial model is good enough to determine the active site; yet, Au and Ag atoms were placed on different bridging rows, so there was no interaction between Au and Ag atoms. Hence, no charge transfer could take place from Ag to Au. To understand the water oxidation mechanism at the interface of noble metal atoms cluster and TiO_2_ metal oxide surface, we further extended calculations to use a 3-dimension tetrahedral cluster of (Au and Ag) atoms on the TiO_2_ surface because of its stability. This structure we refer to as a cluster model. Our previous work^34^ showed that the Au_2_–Ag_2_/TiO_2_ structure is more suitable when embedded in the location of oxygen vacancy. Nevertheless, we show how the OER activity is affected when the tetrahedral cluster is placed on a stoichiometric surface at the two bridging oxygen rows (a different position), as shown in Fig. 8c. In the supplementary document, we have demonstrated OER intermediates of Au_4_/TiO_2_, Ag_4_/TiO_2_, and Au_2_–Ag_2_/TiO_2_ when placed on two bridging rows (see figure S5 and S6). The comparative free energy diagram for Au_4_/TiO_2_, Ag_4_/TiO_2_ on two bridging rows is given in figure S9 (a) and (b) with the OER data (relative free energies) shown in table S3.

**Figure 4:**
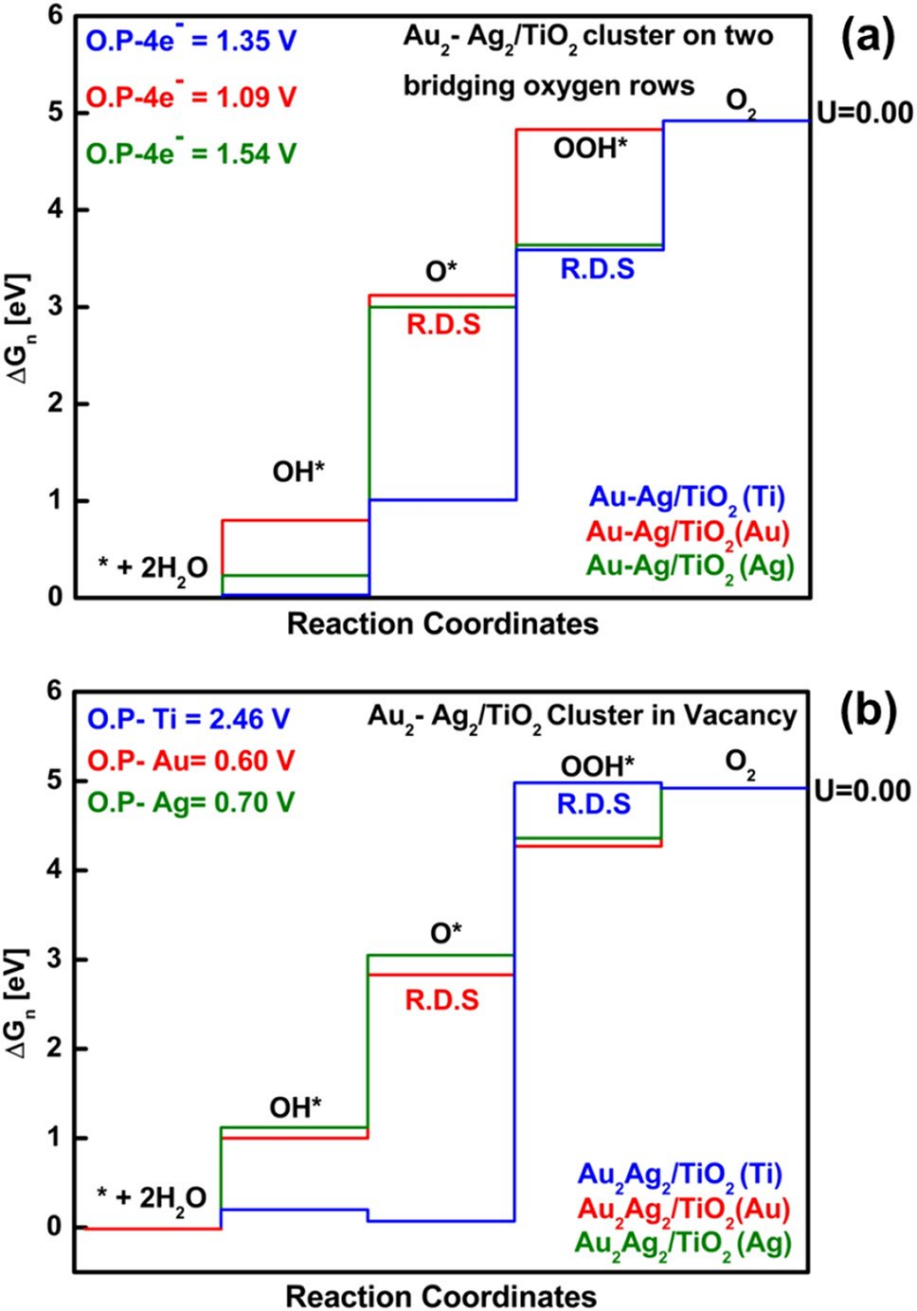
Comparative free energy diagrams for the evolution of O_2_ on Au_2_-Ag_2_ cluster (**a**) placed on two bridging oxygen atoms, (**b**) embedded in the place of oxygen vacancy on bridging row^34^. The blue profile shows the energy for Ti active site, Green and red color shows energy profiles for Ag and Au active sites, respectively. The overpotential is shown in the upper left corner. *R.D.S* rate-determining step.

Figure 4a shows the results for Au_2_–Ag_2_/TiO_2_ on two different bridging rows. The comparative free energy profile shows that the lowest overpotential is when Au was chosen as an active site. However, the OH*, O*, and OOH* steps were strongly bonded for the Ti active site than Au and Ag. Similar findings were observed for the model when Au_2_–Ag_2_/TiO_2_ was placed on a bridging row oxygen vacancy. The O_2_ evolution was preferred on the Au site, free energy diagram shown in Fig. 4b. From the above-shown results, it has been seen that either Au_2_–Ag_2_ cluster adsorb on TiO_2_ surface by connecting to two bridging oxygen rows or an oxygen vacancy is created, and then the cluster is anchored into the vacant site; the perfect active site is Au. In all cases, Ti is the least preferred due to high overpotential. It was also found that the rate-determining step for OER on Au_2_–Ag_2_/TiO_2_ surface is the O* intermediate, i.e., 2nd step when the active site is noble metal atoms, i.e., Au or Ag. While the rate-determining step for OER with Ti as an active site is OOH* intermediate, i.e., the 3rd step. For Au_2_–Ag_2_/TiO_2_, the perfect active site is Au. When applied bias (U = 0 V), all steps go uphill thermodynamically.

When Au_2_–Ag_2_/TiO_2_ cluster is adsorbed on two bridging rows, TiO_2_ surface is stoichiometric; however, a remarkable change is noticed in overpotential when embedded on a vacant oxygen site. This change is attributed to the presence of oxygen vacancy because Au and Ag atoms are higher in electronegativity and prefer to stay in the place of oxygen vacancy. Therefore, there is excess electron density when the oxygen atom from the bridging row is removed. This "excess electron density" attaches the silver and gold clusters (with the ability of electronegativity) to the vacancy site. Moreover, when gold and silver atoms are bonded together in bimetallic clusters, there is a charge transfer from Ag to Au, which is accountable for making Au negatively charged and expected to be more active.

For the cluster systems, the relative free energies of all the intermediates (OOH*, OH*, and O*) relative to the starting state (* + 2H_2_O) are shown in Table 2. For all systems, we see that the trend in activity is the same for the active site, i.e., Au > Ag > Ti. For noble metal atoms, the rate-determining step is the O* step, and for transition metal, the rate-determining step is OOH*. Notably, the OOH* step is much less stable when the active site is Ti (blue curves in free energy diagrams) conversely the OH* and O* are more stable and vice versa for the noble metal active sites case (red and green curve for Au and Ag respectively in free energy diagrams).

**Table 2:** The relative free energies for each type of active sites on the Au_2_–Ag_2_/TiO_2_ with a cluster on two bridging oxygen rows and bridging row oxygen vacant site shown in Figs. 8c and 9c, respectively, starting from the dissociation of a water molecule (H_2_O + * ↔ H* + HO*) set as the reference point with zero energy.

Active site	ΔG_OH*_	ΔG_O*_	ΔG_OOH*_	Overpotential (V)	Rate-determining steps
**System: Au**_**2**_**–Ag**_**2**_**/TiO**_**2**_** with cluster on two bridging oxygen rows**					
Au	0.80	3.12*	4.83	1.09	O* + H_2_O → HOO* + ½H_2_
Ag	0.23	3.00*	3.64	1.54	O* + H_2_O → HOO* + ½H_2_
Ti	0.03	1.01	3.59*	1.35	HOO* → O_2_ + ½H_2_
**System: Au**_**2**_**–Ag**_**2**_**/TiO**_**2**_** cluster on bridging row oxygen vacant site**					
Au	1.00	2.83*	4.27	0.60	O* + H_2_O → HOO* + ½H_2_
Ag	1.12	3.05*	4.36	0.70	O* + H_2_O → HOO* + ½H_2_
Ti	0.20	0.07	3.76*	2.46	HOO* → O_2_ + ½H_2_

The propagating step is denoted as ΔG_OH*_, ΔG_O*_, and ΔG_OOH*_ shown together with their overpotentials and rate-determining step. *The rate-determining steps.

### OER performance of TiO_2_ supported Ag and Au catalysts

**Figure 5:**
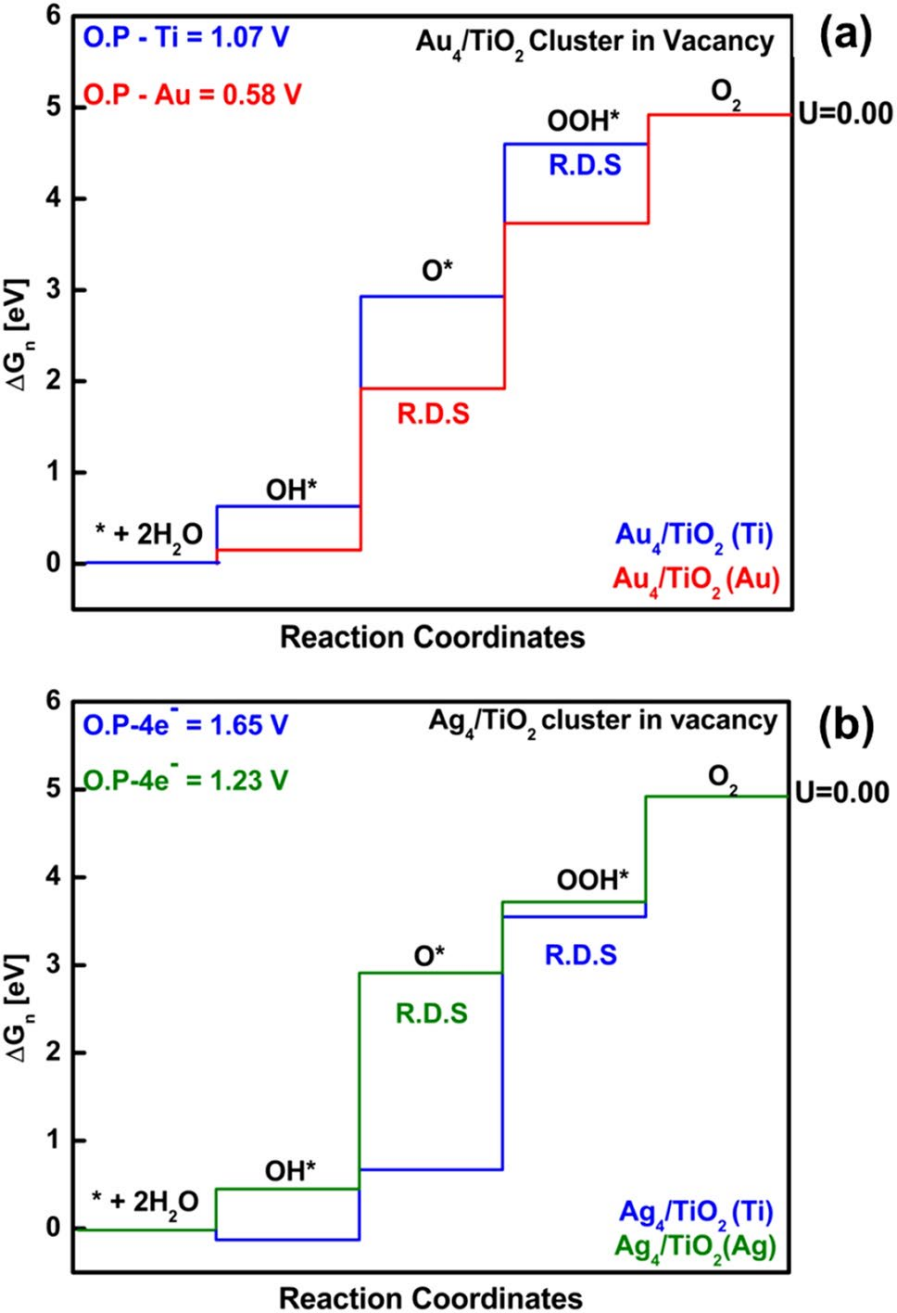
Comparative free energy diagram for (**a**) Ag_4_/TiO_2_ and (**b**) Au_4_/TiO_2_. Overpotential is shown in the upper left corner. The rate-determining step (R.D.S) for Ag and Au as active sites is O* while OOH* for Ti atom as an active site. Thus, the blue profile represents Ti as an active site, the red profile represents Au as an active site, and the green profile represents Ag as an active site.

As explained earlier, adsorption of Ag on TiO_2_ surface becomes more stable when interacted with Au. Moreover, the photocatalytic property of Ag species is enhanced because Au promotes charge transfer from Ag to Au on the TiO_2_ surface. To explain this phenomenon in-depth, we aimed to study the effect of only Au on TiO_2_ and Ag on TiO_2_ with cluster and initial model. We used the same strategy for choosing the active site and stable structure mentioned earlier for the bimetallic cluster. However, we only discuss the results of Au4/TiO_2_ and Ag4/TiO_2_ cluster models shown in Fig. 10a,b, and Table 3﻿, respectively. Free energy diagrams are shown in Fig. 5a,b and for OER intermediates (see S.I. figure S7). For Au_4_/TiO_2_ and Ag_4_/TiO_2_ clusters placed on two bridging rows, free energy diagrams are given in S.I figure S9.

**Table 3:** The relative free energies for each type of active sites on the monometallic Au_4_/TiO_2_ and Ag_4_/TiO_2_ cluster bridging row oxygen vacant site shown in Fig. 7a,b, starting from the dissociation of a water molecule (H_2_O + * ↔ H* + HO*) set as the reference point with zero energy.

Active site	ΔG_OH*_	ΔG_O*_	ΔG_OOH*_	Overpotential (V)	Rate-determining steps
**System: Au**_**4**_**/TiO**_**2**_**, replacing bridging O atoms on the surface with Au**_**4**_** cluster in vacancy**					
Au	0.15	1.92	3.73*	0.58 V	O* + H_2_O → HOO* + ½H_2_
Ti	0.63	2.93*	4.60	1.07 V	HOO* → O_2_ + ½H_2_
**System: Ag**_**4**_**/TiO**_**2**_**, replacing bridging O atoms on the surface with Ag**_**4**_** cluster in vacancy**					
Ag	0.45	2.91*	3.72	1.23 V	O* + H_2_O → HOO* + ½H_2_
Ti	− 0.13	0.67	3.55*	1.65 V	HOO* → O_2_ + ½H_2_

The propagating step is denoted as ΔG_OH*_, ΔG_O*_, and ΔG_OOH*_ shown together with their overpotentials and rate-determining step. *The rate-determining steps.

Studying the OER mechanism on all Au and Ag clusters, i.e., Ag_4_/TiO_2_ and (b) Au_4_/TiO_2_, placed in bridging row oxygen vacancy, we found that overpotential for Ag active site was 0.53 V higher for Ag_4_/TiO_2_ when compared with Ag_2_–Au_2_/TiO_2_, i.e., 0.70 V. However, for the Ti active site, overpotential is remarkably less, and a difference of 0.81 V was observed. If we compare Au_4_/TiO_2_ with Ag_2_–Au_2_/TiO_2_ with Au active site, the overpotential remains the same almost. However, there are noticeable changes for the Ti active site, i.e., 1.39 V. So we conclude from our results that, in the case of any model, either initial or cluster, the active site is the preferably Au atom. In terms of overpotential, the trend of photocatalytic activity on all studied models is Au > Ag > Ti.

### Insights into OER

We mentioned earlier that Ag noble metal could be an excellent approach over Au for plasmonic photocatalytic activity; however, the plasmonic frequency of Ag is near the UV region responsible for limiting the catalytic activity of Ag-based photocatalyst. Therefore, we make a bimetallic alloy of Au and Ag. From our OER calculations, we have concluded that on Ag_4_/TiO_2_ over potential is higher for Ag active site, i.e., 1.23 V, for Au_4_/TiO_2_ overpotential is lower with Au active site, i.e., 0.58 V. But when Au and Ag are in a bimetallic cluster state, and we perform OER calculations on Au_2_–Ag_2_/TiO_2_, the overpotential with Au and Ag active site is 0.60 V and 0.70 V, respectively. Hence, this proves that the presence of Au noble metal could stabilize the Ag and lead to better photocatalytic activity. Unfortunately, there are fewer reports of OER studies on noble metal-loaded TiO_2_ structures. Wang et al.^57^ reported the OER mechanism on Au/TiO_2_ initial structure. Instead of removing Bridging oxygen, surface oxygen was removed and was further doped with Au atom. They only chose Ti as an active site. The rate-determining step was OOH* with an overpotential of 1.77 V. To provide further insight about noble metal-doped TiO_2_ surface, the calculations were extended by using a small (four atoms) tetrahedral cluster for determining the OER activities. In the near future, studies with larger bimetallic clusters are needed to check if the trends observed here stay within the range of bimetallic particles.

### Bader charge analysis

Oxygen vacancy is one of the vital defects among all the defects known in TiO_2_^58^. It has been discovered that oxygen vacancies can perform as significant adsorption and active sites for heterogeneous catalysis, which can sturdily sway the reactivity of metal oxides^59^. Theoretically, oxygen vacancies formation on TiO_2_ is direct to the creation of unpaired electrons or Ti^3+^ centers, which possibly will form donor levels in the electronic structure of TiO_2_^60^. Theoretical and experimental results indicate that the excess electrons are located on the oxygen vacancy states. When noble metal atoms (Au or Ag) are adsorbed on the TiO_2_ surface, they tend to adsorb on the oxygen vacant site due to the high electronegativity of noble metal atoms.

**Figure 6:**
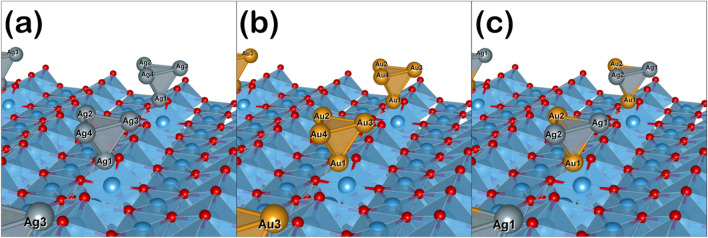
Configurations of the TiO_2_-supported monometallic and bimetallic catalysts of (**a**) Ag_4_/TiO_2_, (**b**) Au_4_/TiO_2_, and (**c**) Au_2_-Ag_2_/TiO_2_. The color code is blue for Ti, red for O, silver for Ag, and gold for Au.

We performed the Bader charge analysis to investigate the electron transfer between the metal active site (Au, Ag, or Ag–Au) and the catalyst surface for Ag_4_/TiO_2_, Au_4_/TiO_2_, and Ag_2_–Au_2_/TiO_2_ system, as shown in Fig. 6. The calculated values are given in Table 4. The negative and positive signs refer to electron accumulation and depletion, respectively.

**Table 4:** Charge of each atom derived from the Bader charge analysis of Ag_4_**/**TiO_2_, Au_4_/TiO_2_ and Ag_2_–Au_2_/TiO_2_ systems.

System	Cluster	Bader charge (|e|)	
Ag_4_/TiO_2_	Ag_4_	Ag_4_ cluster	+ 0.14
		Ag1 atom	− 0.23
		Ag2 atom	+ 0.10
		Ag3 atom	+ 0.04
		Ag4 atom	+ 0.23
Au_4_/TiO_2_	Au_4_	Au_4_ cluster	− 0.32
		Ag1 atom	− 0.41
		Ag2 atom	+ 0.01
		Ag3 atom	− 0.05
		Ag4 atom	+ 0.13
Ag_2_–Au_2_/TiO_2_	Ag_2_–Au_2_	Ag_2_–Au_2_ cluster	− 0.14
		Au_2_ subcluster	− 0.69
		Ag_2_ subcluster	+ 0.55
		Au1 atom	− 0.48
		Au2 atom	− 0.21
		Ag1 atom	+ 0.22
		Ag2 atom	+ 0.33

For the Ag_4_/TiO_2_ structure, the positive value of the Ag_4_ cluster (+ 0.14 |e|) designates its role as an electron donor during its adsorption on the TiO_2_ surface with an oxygen vacancy site. Moreover, a negative Bader charge of Ag1 (− 0.23 |e|) together with the positive Bader charge of other surrounding Ag atoms (varies from + 0.04 to + 0.23 |e|) reveal electron transfer from the oxygen vacancy site to other Ag atoms: Ag2, Ag3, and Ag4, to Ag1 atom. Hence, the activity of the Ag1 atom is improved.

For Au_4_/TiO_2_ structure, the Au_4_ cluster plays a role as an electron acceptor, taking electrons from the TiO_2_ surface. Similar to the Ag_4_/TiO_2_ structure, the most negative Bader charge is found on the Au1 site, which specifies that the activity of the Au1 site is enhanced after its adsorption on the TiO_2_ surface with oxygen vacancy site. In addition, the electron accumulations around the Au1 site is found to be two times higher than that of the Ag1. This suggested a high activity of the Au system.

For the Ag_2_–Au_2_/TiO_2_ system, the whole bimetallic Ag_2_–Au_2_ cluster acted as an electron acceptor similar to the Ag cluster in the Ag_4_/TiO_2_ system. However, the total Bader charge value of the Ag_2_–Au_2_ cluster is less negative (− 0.14 |e|). Considering the two-atom Ag (Ag_2_) and two-atom Au (Au_2_) cluster within the Ag_2_–Au_2_ cluster, it was found that the Au_2_ subcluster holds a negative Bader charge of − 0.69|e|, while the positive Bader charge was found in the Ag_2_ subcluster of + 0.55 |e|. This phenomenon describes the additional electron transfer from the Ag_2_ to the Au_2_ subcluster.

In addition, the activity of the Au1 active site in the Ag_2_–Au_2_ cluster is promoted via the electron transfer from the Ag site. Moreover, the Au1 site located at the vacancy site of the TiO_2_ surface still has the most negative Bader charge value of − 0.48 |e|. This Bader charge value of the Au1 atom in the Ag_2_–Au_2_ cluster is similar to the Au1 atom in the Au_4_ cluster. Hence, the presence of Ag species in the Ag_2_–Au_2_ bimetallic cluster facilitates electron delocalization around Au1 and Au2, creating another active site—the Au2 site. Therefore, the activity of the bimetallic Ag_2_–Au_2_ cluster exceeds the monometallic cluster of Au4 and Ag4 clusters due to the presence of the Ag_2_ subcluster, which donates additional electrons to the Au_2_ subcluster.

## Conclusion

In summary, we used density functional theory to investigate the role of Au metal in Au–Ag high noble alloys catalysts supported on TiO_2_ on the performance during the oxygen evolution of water oxidation (OER), in which the catalysts are modeled as Au, Ag, and Au–Ag supported on rutile TiO_2_ (110). Since doping of noble metal atoms increases the photocatalytic activity of TiO_2_, the Ag noble metal is shown to have a promising catalytic activity among the groups. However, studying the reaction mechanism on such a surface is essential due to its low stability.

Combining Au (a more stable noble atom) into Ag-based TiO_2_ forming bimetallic high-noble alloy catalysts can enhance photocatalytic activity. The study investigated the high-noble alloy cluster in terms of the most stable configuration verified by the most stable position of the metal-support interface, stability of the active site, optimal size, and the most active region of the catalyst for the OER during water oxidation. The most stable location of the metal-support interface for the Au–Ag high-noble alloy catalyst modeled by the Au_2_–Ag_2_/TiO_2_ was found at the bridging row oxygen vacant site. On the performance of this high-noble alloy catalyst, the Au atom is always preferred as an active site for OER regardless of the size or position of the cluster. The photocatalytic activity trend indicated by an overpotential and active site preference is Au > Ag > Ti. Hence, in both cases of monometallic (Au and Ag) and bimetallic (Au–Ag), the Ti active site is least preferred.

With this positioning, the overpotential is much lower for the Au and Ag active site, i.e., 0.60 V and 0.70 V, indicating that the Au atom stabilizes Ag. Therefore, utilizing the high-noble alloy catalyst of Au–Ag can improve the oxygen evolution activity on the rutile TiO_2_ (110) surface during the water oxidation reaction, promoting efficient hydrogen production and supporting the hydrogen economy.

In addition, the future design of high photocatalytic performance catalyst based on this study must consider the profiles from the Bader charge analysis, which suggested that the presence of the Ag atom can stabilize the Au atom via the electron transfer to the Au, where this generated the trap state between the valence band maximum (VBM) and the conduction band minimum (CBM) reducing the band gap of the catalyst promoting activity as supported by the projected density of states (DOS) profile of this Au–Ag/TiO_2_ catalyst.

## Methods

### Computational details

#### Density functional theory-based calculations

We employed the first-principles spin-polarized density functional theory (DFT) calculations using the projector augmented wave (PAW) method applied in the Vienna Ab initio simulation package (VASP)^61,62^. For the exchange–correlation functional, the generalized gradient approximation (GGA)^63^ of Perdew − Burke − Ernzerhof (PBE)^64^ was used with the plane-wave cutoff energy of 400 eV. Ultra-soft pseudo-potential, the interaction between valence electrons and the ionic core^65^, is used with 2s^2^ 2p^4^, 3d^2^ 4s^2^,4d^10^5s^1^, 5d^10^6s^1^ as the valence electrons configuration for the O, Ti, Ag, and Au atoms, respectively. The Monkhorst and Pack scheme of k-point sampling was employed to integrate the first Brillouin zone^66^, and a 3 × 2 × 1 grid was used to obtain the geometry optimization and total energies for the rutile TiO_2_ (110) surface. The applied residual forces for geometry optimization and convergence on the atoms were 0.01 eV/Å, and the self-consistent iteration awaits the tolerance for total energy to get to 10–4 eV. The optimized lattice parameters a = 11.83 Å, b = 12.99 Å, c = 30.08 Å, and α = β = γ = 90.00° for rutile (110). The calculations were done on a four-layered rutile TiO_2_ (110) surface slab with a super-cell of 3 × 2 × 1 where the bottom layer was fixed at bulk-truncated positions and the others were fully relaxed. Dipole correction was applied to all of the calculations. We calculated the energy position, valence band maximum (VBM), conduction band minimum (CBM), and trap states level for band structure calculations. For these properties, we carried out single-point energy calculations with the HSE06 functional^67^ because they are known to provide a better description of the valence band (VB) and conduction band (CB), band edges, and band gaps (that are underestimated with PBE).

### Catalyst model constructions

**Figure 7:**
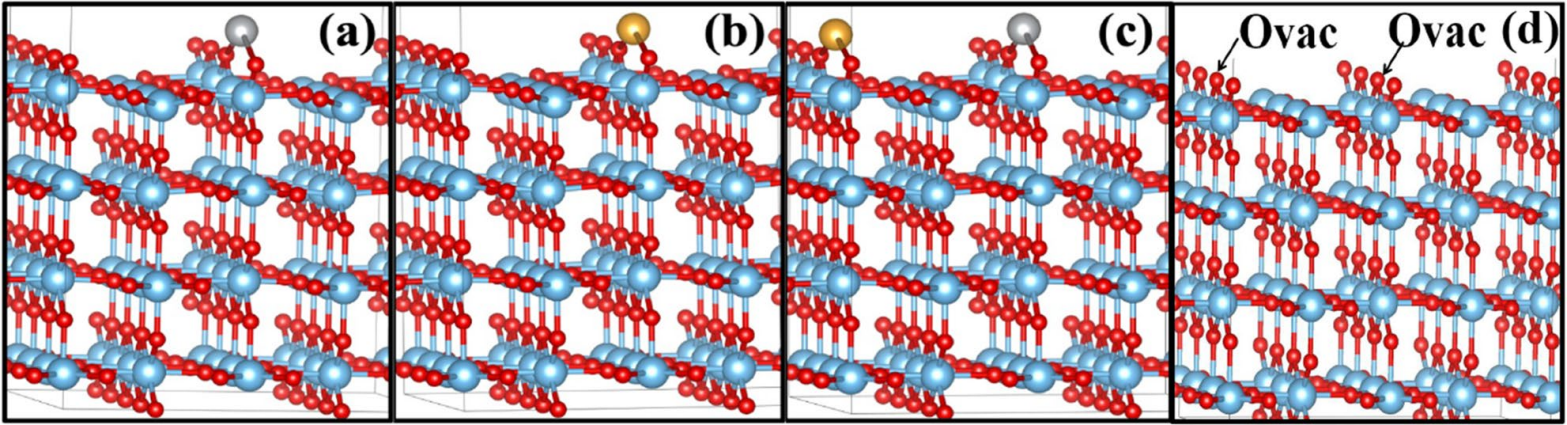
Initial model of one atom (**a**) Ag on TiO_2_, (**b**) Au on TiO_2_, (**c**) Au–Ag on TiO_2_ (d) clean rutile TiO_2_ (110) surface model.

**Figure 8:**
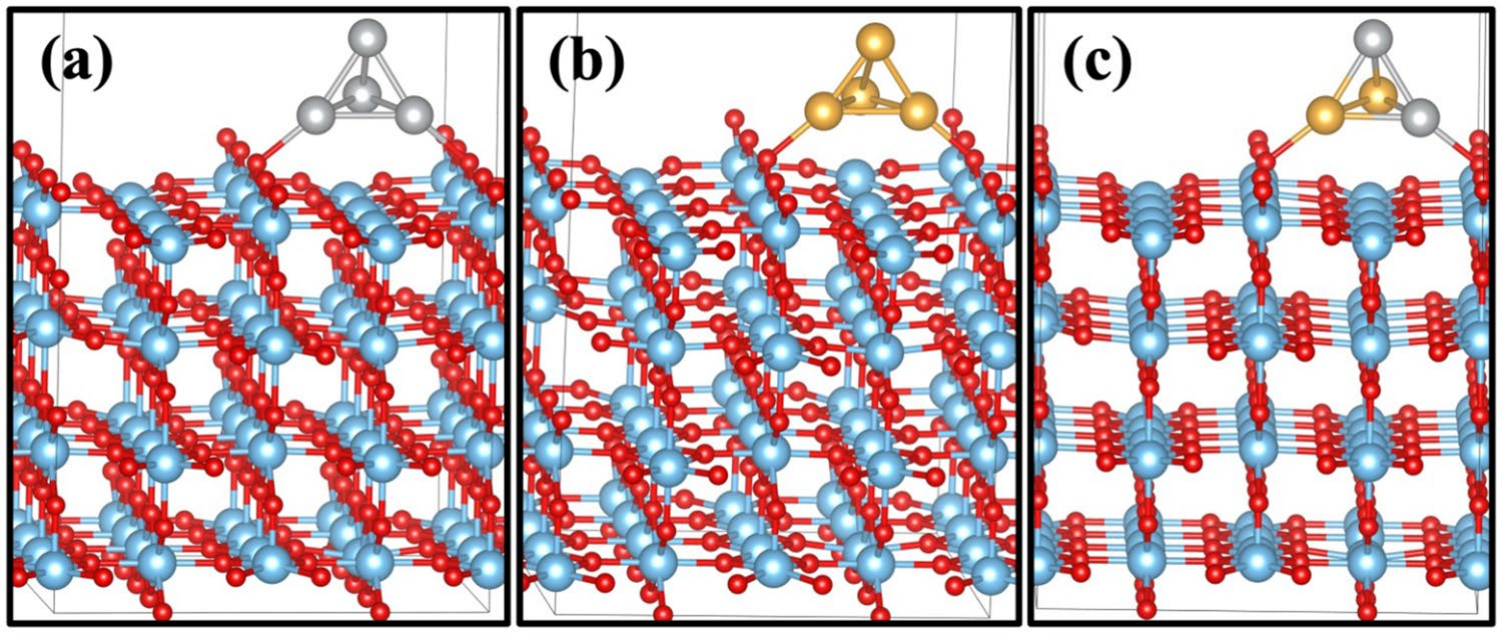
Four atoms cluster on two bridging rows of (**a**) Ag_4_ on TiO_2_, (**b**) Au_4_ on TiO_2_, and (**c**) Ag_2_–Au_2_ on rutile TiO_2_ (110) surface model.

**Figure 9:**
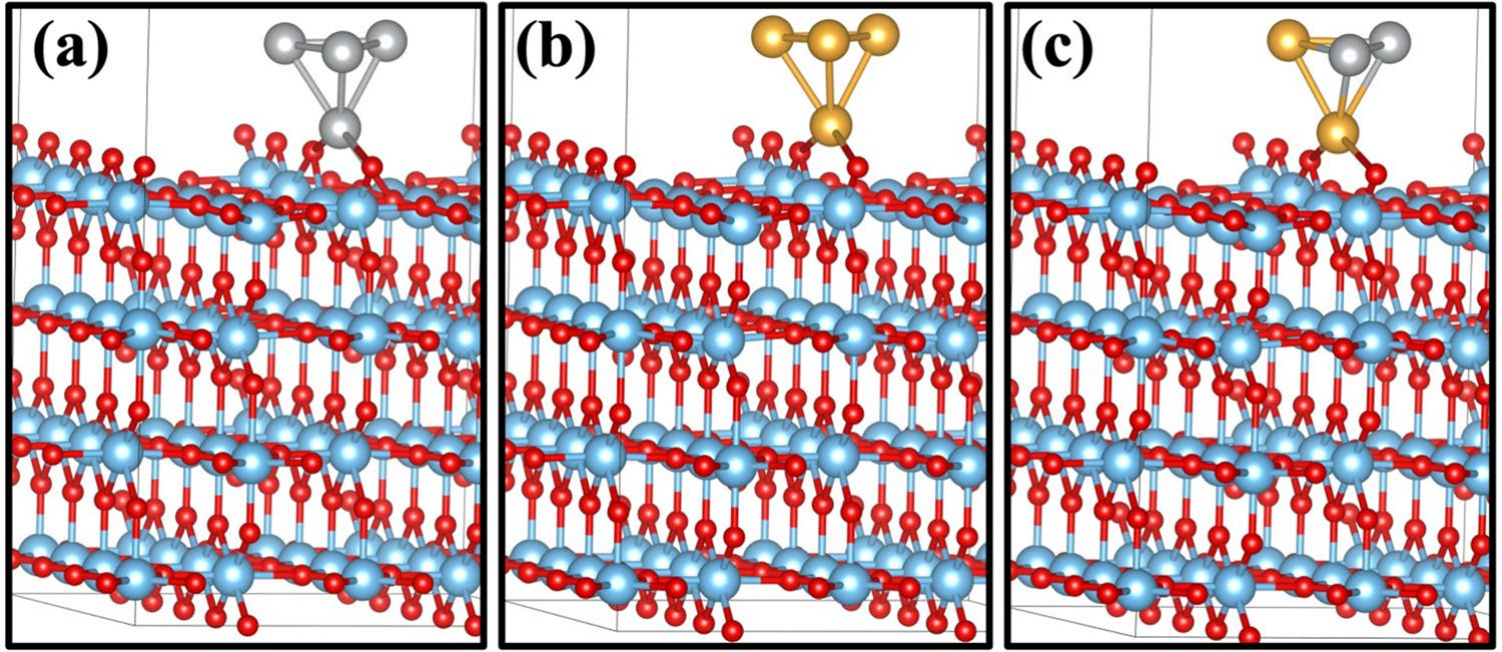
Four atoms cluster on bridging row oxygen vacant site of (**a**) Ag_4_ on TiO_2_, (**b**) Au_4_ on TiO_2_, and (**c**) Ag_2_–Au_2_ on rutile TiO_2_ (110) surface model.

The OER mechanism was studied on the Au/TiO_2_, Ag/TiO_2_, and Au–Ag/TiO_2_. For the initial model: We created an oxygen vacancy in the bridging row on the surface which was filled by gold atom in Au/TiO_2_ model (Fig. 7a) and similarly Ag atom for Ag/TiO_2_ (Fig. 7b); however, in the case of the Au–Ag/TiO_2_ model, we replaced two bridging oxygen atoms from different bridging rows on the surface to replace Au and Ag atoms shown in Fig. 7c. Figure 7d shows the clean rutile (110) surface. We have demonstrated the bridging oxygen, which was pulled out to create oxygen vacancies. These configurations are shown to be at the most stable metal-support interface location. For the cluster model: We placed the tetrahedral cluster of 4 atoms Au_4_, Ag_4_, and Au_2_–Ag_2_ with different orientations on the surface, as shown in Figs. 8 and 9. We chose the cluster as a model because of its stability^68^. The result exhibited that the Au_2_–Ag_2_ alloy anchored on bridging oxygen on top of the rutile TiO_2_ (110) surface is the most stable, wherein Fig. 7d, a clean surface, is shown with the chosen oxygen vacancies marked as O_vac_.

### Evaluation of water oxidation performance

The reaction mechanism of water oxidation can occur with four proton-coupled electron transfer steps, where the initial step is the water adsorption process. Our calculations showed that water molecules tend to adsorb on the TiO_2_ surface. We used the mechanism proposed by Rossmeisl et al.^69^. The OER mechanism is progressed in the following four steps as shown in equations 1–4, where ½ H_2_ ↔ H^+^  + *e*^−^ is the half electrode reaction.

1 $${\text{H}}_{{2}} {\text{O}} + * \to {\text{HO}} * + \raise.5ex\hbox{$\scriptstyle 1$}\kern-.1em/ \kern-.15em\lower.25ex\hbox{$\scriptstyle 2$} {\text{ H}}_{{2}}$$

2 $${\text{HO}} * \to {\text{O}} * + \, \raise.5ex\hbox{$\scriptstyle 1$}\kern-.1em/ \kern-.15em\lower.25ex\hbox{$\scriptstyle 2$} {\text{H}}_{{2}}$$

3 $${\text{O}} * + {\text{H}}_{{2}} {\text{O}} \to {\text{HOO}} * + \, \raise.5ex\hbox{$\scriptstyle 1$}\kern-.1em/ \kern-.15em\lower.25ex\hbox{$\scriptstyle 2$} {\text{H}}_{{2}}$$

4 $${\text{HOO}} * \to {\text{O}}_{{2}} + \raise.5ex\hbox{$\scriptstyle 1$}\kern-.1em/ \kern-.15em\lower.25ex\hbox{$\scriptstyle 2$} {\text{H}}_{{2}}$$

**Figure 10:**
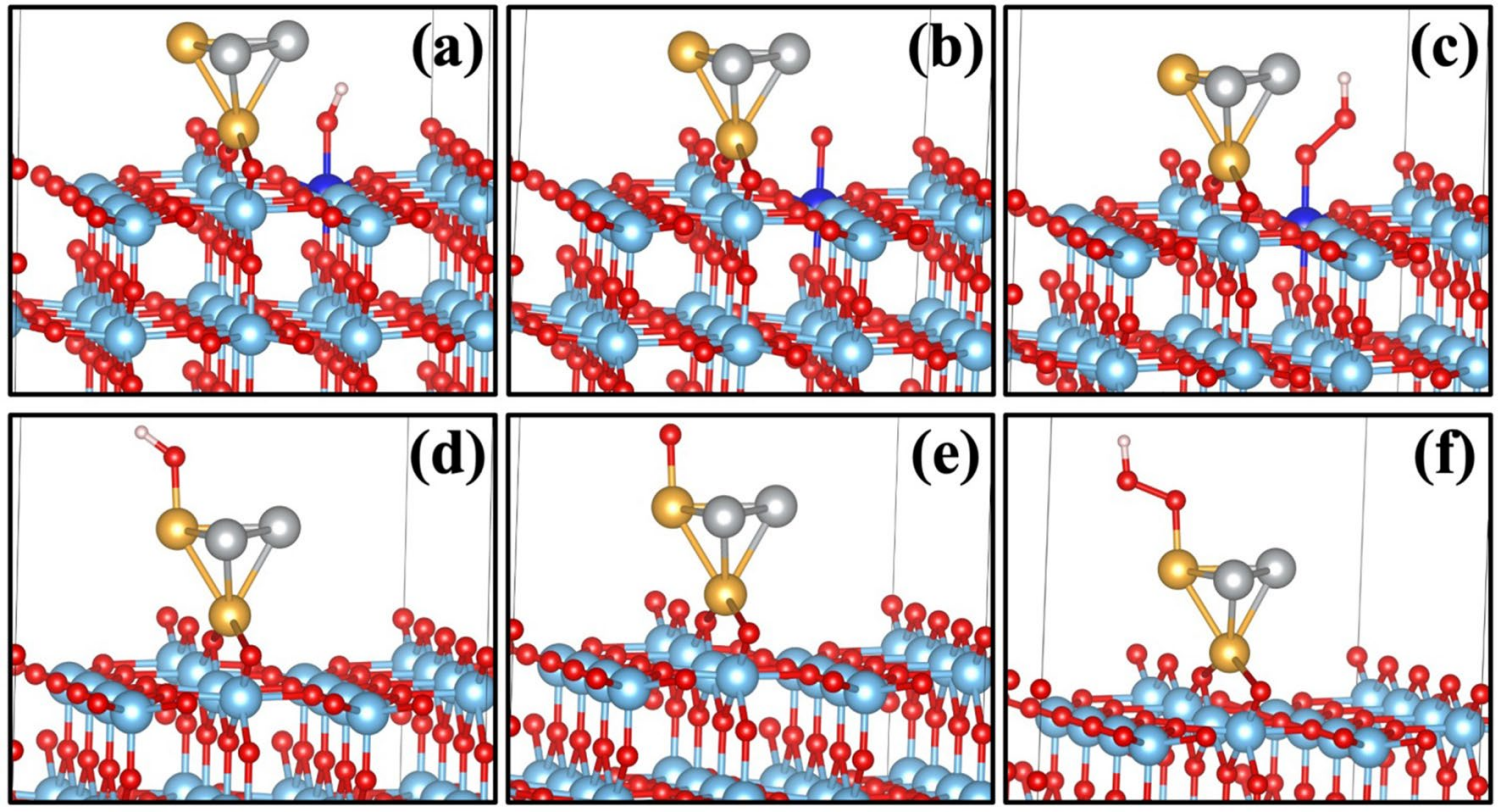
OER intermediates on Au_2_–Ag_2_/TiO_2_ semiconductor surface with Ti active site marked blue in (**a**) OH*-Ti, (**b**) O*-Ti, (**c**) OOH*-Ti and with gold as active site shown in (**d**) OH*-Au, (**e**) O*-Au, and (**f**) OOH*-Au.

The OER intermediates are shown in Fig. 10a–f on Ti and Au as an active site, for Ag active site (see figure S2) in S.I. To evaluate the steps as mentioned above, it is expedient to characterize the free energies ΔG_OH*_, ΔG_O*_ and ΔG_OOH*_ of the OH*, O*, OOH* intermediate states, and ΔG_O2_ for the O_2_ final stage, all determined relative to the resting state (* + 2H_2_O). In Sect. 1 of supplementary information (S.I), we have details of the determination of the free energies (including vibrational and entropy corrections). We can extract the reaction step-free energies from the relative free energies; ΔG_n_ (n = I, II, III, and IV) are written as equations 5–6.

5 $$\Delta {\text{G}}_{{\text{I}}} = \, \Delta {\text{G}}_{{{\text{OH}}*}}$$

6 $$\Delta {\text{G}}_{{{\text{II}}}} = \Delta {\text{G}}_{{{\text{O}}*}} - \Delta {\text{G}}_{{{\text{OH}}*}}$$

7 $$\Delta {\text{G}}_{{{\text{III}}}} = \Delta {\text{G}}_{{{\text{OOH}}*}} - \, \Delta {\text{G}}_{{{\text{O}}*}}$$

8 $$\Delta {\text{G}}_{{{\text{IV}}}} = \Delta {\text{G}}_{{{\text{O2}}}} - \Delta {\text{G}}_{{{\text{OOH}}*}}$$

The overpotential of reactions could be associated reliably to the proton and electron transfer to adsorbed species strongly bonded to the surface at the electrode potential. This reaction series starts with (* + 2H_2_O) on a metal active site leading to OH*, shown in steps I–IV. The diffusion of species and other surface reactions most likely depends weakly on the potential.

The theoretical overpotential is defined as:

9 $$\upeta = {\text{max }}[\Delta {\text{G}}_{{\text{I}}} ,\Delta {\text{G}}_{{{\text{II}}}} ,\Delta {\text{G}}_{{{\text{III}}}} ,\Delta {\text{G}}_{{{\text{IV}}}} ]/{\text{e}}{-}{1}.{23}\;\left[ {\text{V}} \right]$$

## Supplementary Information

Supplementary Information.

## Data Availability

All data generated or analyzed during this study are included in this published article (and its Supplementary Information files).
