# Supplementary material for: On a high photocatalytic activity of high-noble alloys Au–Ag/TiO2 catalysts during oxygen evolution reaction of water oxidation
Source: Sci Rep. 2022 Feb 16;12:2604. doi: 10.1038/s41598-022-06608-7 (PMC8850597; doi:10.1038/s41598-022-06608-7)
Supplement: Supplementary file 1 — Supplementary Information. [file 41598_2022_6608_MOESM1_ESM.pdf]

On a high photocatalytic activity of high-noble alloys Au-Ag/TiO_2 catalysts during oxygen evolution reaction of water oxidation

Anum Shahid Malik[1,2], Taifeng Liu[3,*], Meena Rittiruam[1,2,4], Tinnakorn Saelee[1,2,5], Juarez L. F. Da Silva[6], Supareak Praserthdam[1,2, *], Piyasan Praserthdam[2]

1. High-Performance Computing Unit (CECC-HCU), Center of Excellence on Catalysis and Catalytic Reaction Engineering (CECC), Chulalongkorn University, Bangkok 10330, Thailand.
2. Center of Excellence on Catalysis and Catalytic Reaction Engineering (CECC), Chulalongkorn University, Bangkok 10330, Thailand.
3. National & Local Joint Engineering Research Center for Applied Technology of Hybrid Nanomaterials, Henan University, Kaifeng, 475004, China.
4. Rittiruam Research Group, Bangkok 10330 Thailand.
5. Saelee Research Group, Bangkok 10330 Thailand.
6. São Carlos Institute of Chemistry, University of São Paulo, PO Box 780, 13560-970 São Carlos, SP, Brazil

[*] Email of corresponding author (Dr. Taifeng Liu): tfliu@vip.henu.edu.cn

[*] Email of corresponding author (Dr. Supareak Praserthdam): supareak.p@chula.ac.th

Keywords: High noble alloys catalysts, rutile TiO_2, water oxidation. oxygen evolution reaction, density functional theory

In the supporting information, it contains

1. The calculation scheme for free energy for oxygen evolution reaction (OER.).
2. Optimized geometries of OER intermediates.
3. Free energy diagrams.
4. Band Structure for Au_4/TiO_2 and Ag_4/TiO_2:
5. Relative free energies tables.

1. **Free energy calculation scheme for oxygen evolution reaction (OER.)**

Thermodynamic potential for water oxidation, $2H_2O \rightarrow O_2 + 4H^+ + 4e^-$, is 4.92 V at standard conditions (T = 298.15 K, P = 1 bar, pH = 0). Nevertheless, a potential of 1.23 V is required in order for this reaction to proceed at a measurable rate. For heterogeneous catalysts, this additional potential is referred to as the overpotential η. To setup the thermo chemistry of the OER, it is more convenient to work at acidic conditions, where steps

$$H_2O + * \rightarrow OH^* + H^+ + e^- \quad (S1)$$

$$OH^* \rightarrow O^* + H^+ + e^- \quad (S2)$$

$$O^* + H_2O \rightarrow OOH^* + H^+ + e^- \quad (S3)$$

$$OOH^* \rightarrow O_2 + H^+ + e^- \quad (S4)$$

The Gibbs free energy change for steps I-IV can be expressed as,

$$\Delta G_I = \Delta G_{OH} - eU + \Delta G_{H^+}(pH) \quad (S5)$$

$$\Delta G_{II} = \Delta G_O - \Delta G_{OH} - eU + \Delta G_{H^+}(pH) \quad (S6)$$

$$\Delta G_{III} = \Delta G_{OOH} - \Delta G_O - eU + \Delta G_{H^+}(pH) \quad (S7)$$

$$\Delta G_{IV} = 4.92\ (eV) - \Delta G_{OOH} - eU + \Delta G_{H^+}(pH) \quad (S8)$$

Where U is the potential measured against normal hydrogen electrode (NHE) at standard conditions (T = 298.15 K, P =1 bar, pH = 0). The Nernst equation represents the free energy change of the protons relative to the above-specified electrode at non-zero pH as ΔG_{H+} (pH) = -kT ln(10) × pH. The sum of ΔG_{I-IV} is fixed to the negative of experimental Gibbs free energy of formation of two water molecules $-2\Delta G_{H2O}$ =4× 1.23 = 4.92 eV to avoid calculating the O_2 bond energy, which is difficult to determine accurately within DFT [1]. The Gibbs free energies of eqs 5-8 depending on the adsorption energies of OH*, O*, and OOH. The Gibbs free energy differences of these intermediates include zero-point energy (Z.P.E.) and entropy corrections accordingly, $\Delta G_i = \Delta E + \Delta ZPE - T\Delta S_i$ and energy differences ΔE_i considered relative to H_2O and H_2 (at U = 0 and pH = 0) as

$$\Delta E_{OH} = E(OH^*) - E(^*) - [E(H_2O) - 1/2\, E(H_2)] \qquad (S9)$$

$$\Delta E_{O} = E(O^*) - E(^*) - [E(H_2O) - E(H_2)] \qquad (S10)$$

$$\Delta E_{OOH} = E(OOH^*) - E(^*) - [2E(H_2O) - 3/2\, E(H_2)] \qquad (S11)$$

The theoretical overpotential is defined as:

$$\eta = \max\, [\Delta G_{I}, \Delta G_{II}, \Delta G_{III}, \Delta G_{IV}]\, /e - 1.23\ [V] \qquad (S12)$$

$\Delta G = \Delta E + \Delta Z.P.E. - T\Delta S$ is calculated as follows: ΔE is reaction energy that we get from density functional theory (DFT). ΔZ.P.E. and ΔS are the differences in zero-point energies and the change in entropy, respectively, obtained from references [2, 3].

Table 1S: Differences in Zero-Point Energies, ΔZ.P.E, and the Change in Entropy ΔS.

**$H_2O + * \rightarrow OH^* + ½ H_2$ $(\Delta Z.P.E. - T\Delta S)_I$**	**$OH^* \rightarrow O^* + ½ H_2$ $(\Delta Z.P.E. - T\Delta S)_{II}$**	**$O^* + H_2O \rightarrow O^* + ½ H_2$ $(\Delta Z.P.E. - T\Delta S)_{III}$**	**$O^* + H_2O \rightarrow O_2 + * + ½ H_2$ $(\Delta Z.P.E. - T\Delta S)_{IV}$**
+ 0.40	- 0.30	+ 0.32	- 0.42

2. Optimized geometries for OER. intermediates:

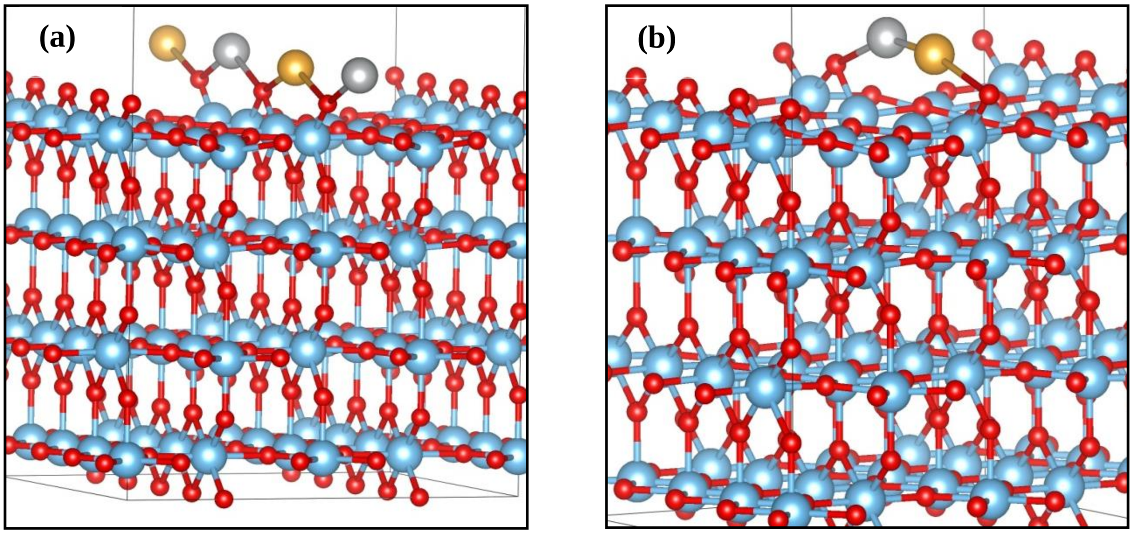

Figure S1: Initial model of Ag-Au/TiO_2 models with least stability ,considered for OER calculations.

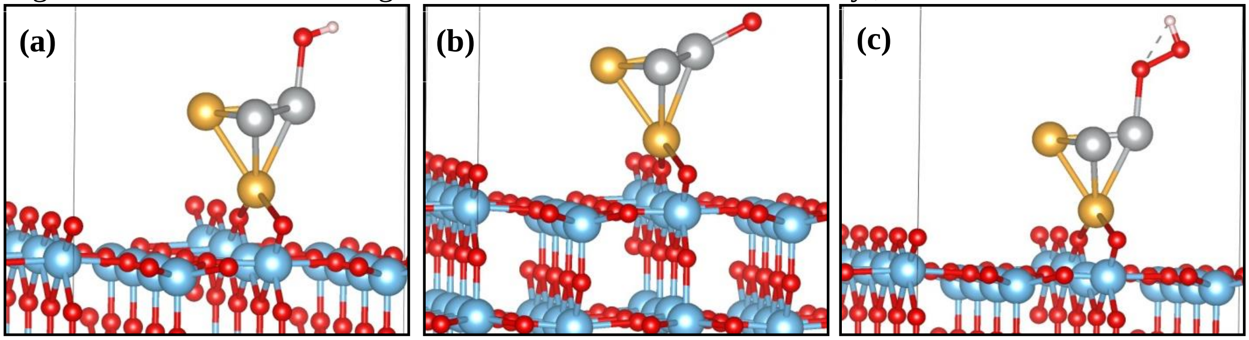

Figure S2: OER optimized intermediates on bridging row oxygen vacant site at ‘Ag’ active site: (a) Ag-OH* (b) Ag-O* (c) Ag-OOH*

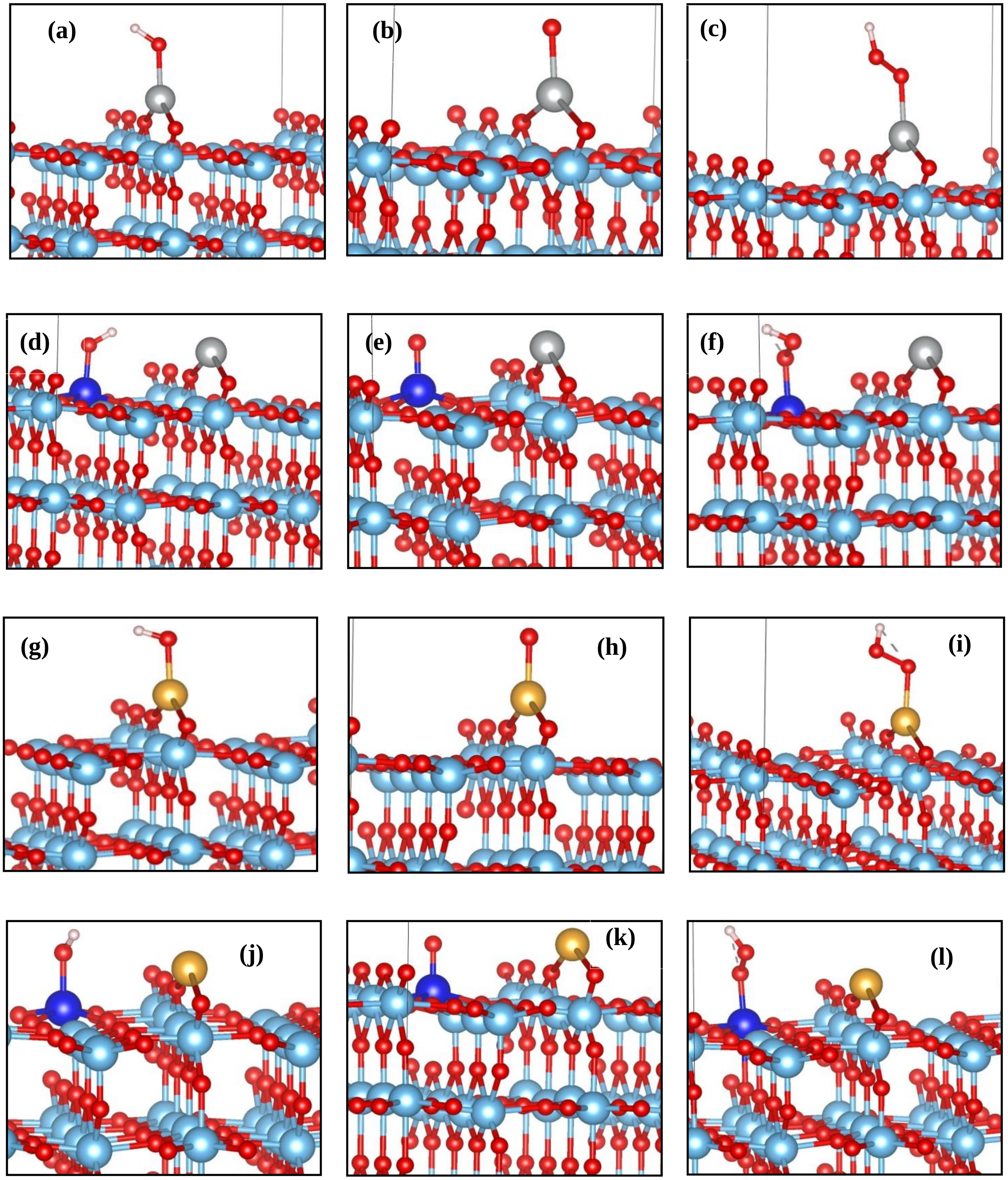

Figure S3: OER optimized intermediates on bridging oxygen vacant site (initial model) Ag/TiO_2 , Ag active site (a) OH*-Ag, (b) O*- Ag (c) OOH*-Ag ; Ti active site (d) OH*-Ti, (e) O*- Ti (f) OOH*-Ti ; Au/TiO_2 , Au active site (g) OH*-Au, (h) O*- Au (i) OOH*- Au ; Ti active site (j) OH*-Ti, (k) O*- Ti (l) OOH*-Ti.

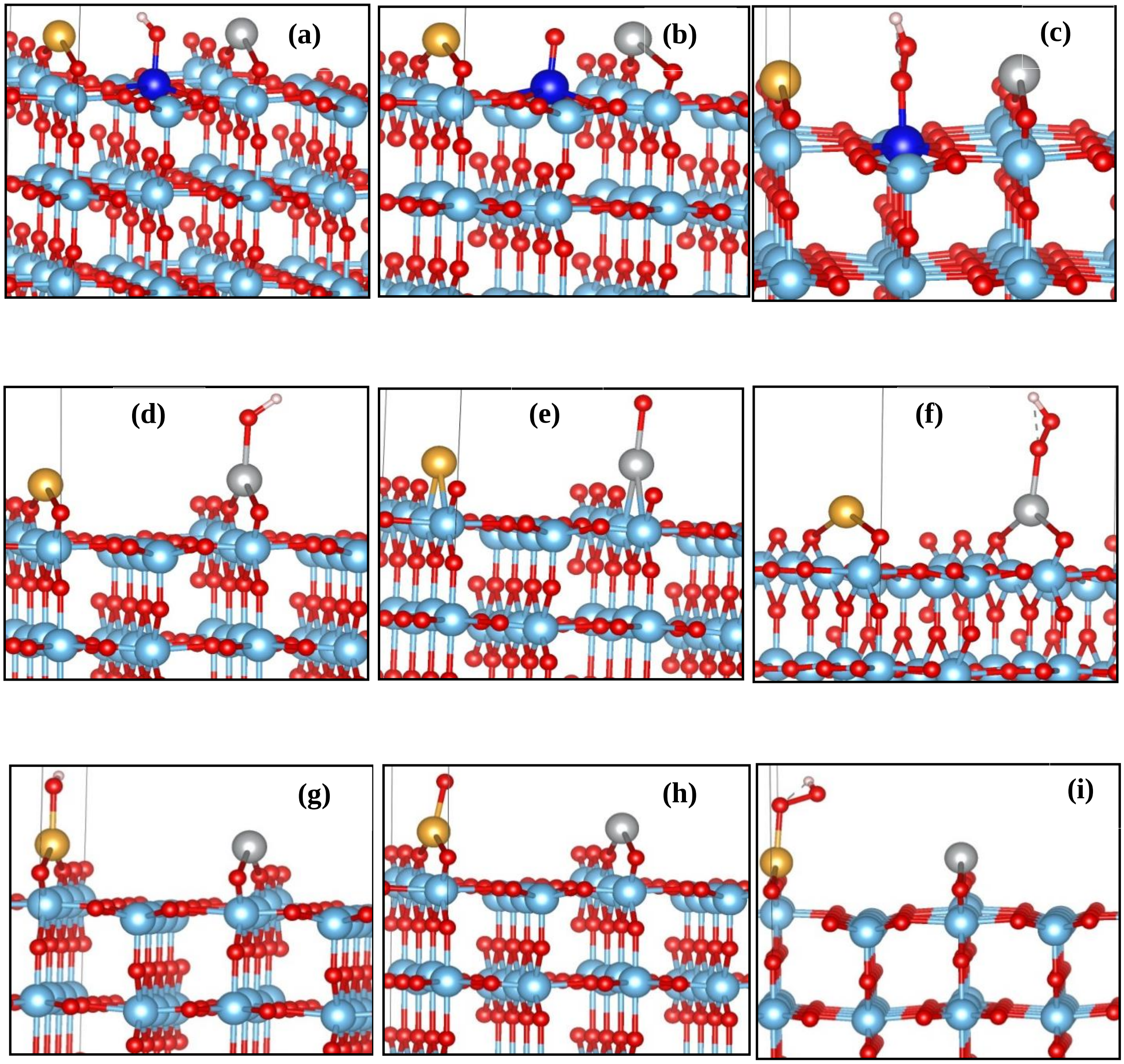

Figure S4: OER optimized intermediates on two bridging rows oxygen vacant sites (initial models) Au-Ag/TiO_2 , Ti active site (a) OH*- Ti, (b) O*- Ti (c) OOH*- Ti. Ag active site (d) OH*-Ag (e) O*- Ag (f) OOH*-Ag ; Au active site (g) OH*-Au (h) O*- Au (i) OOH*-Au.

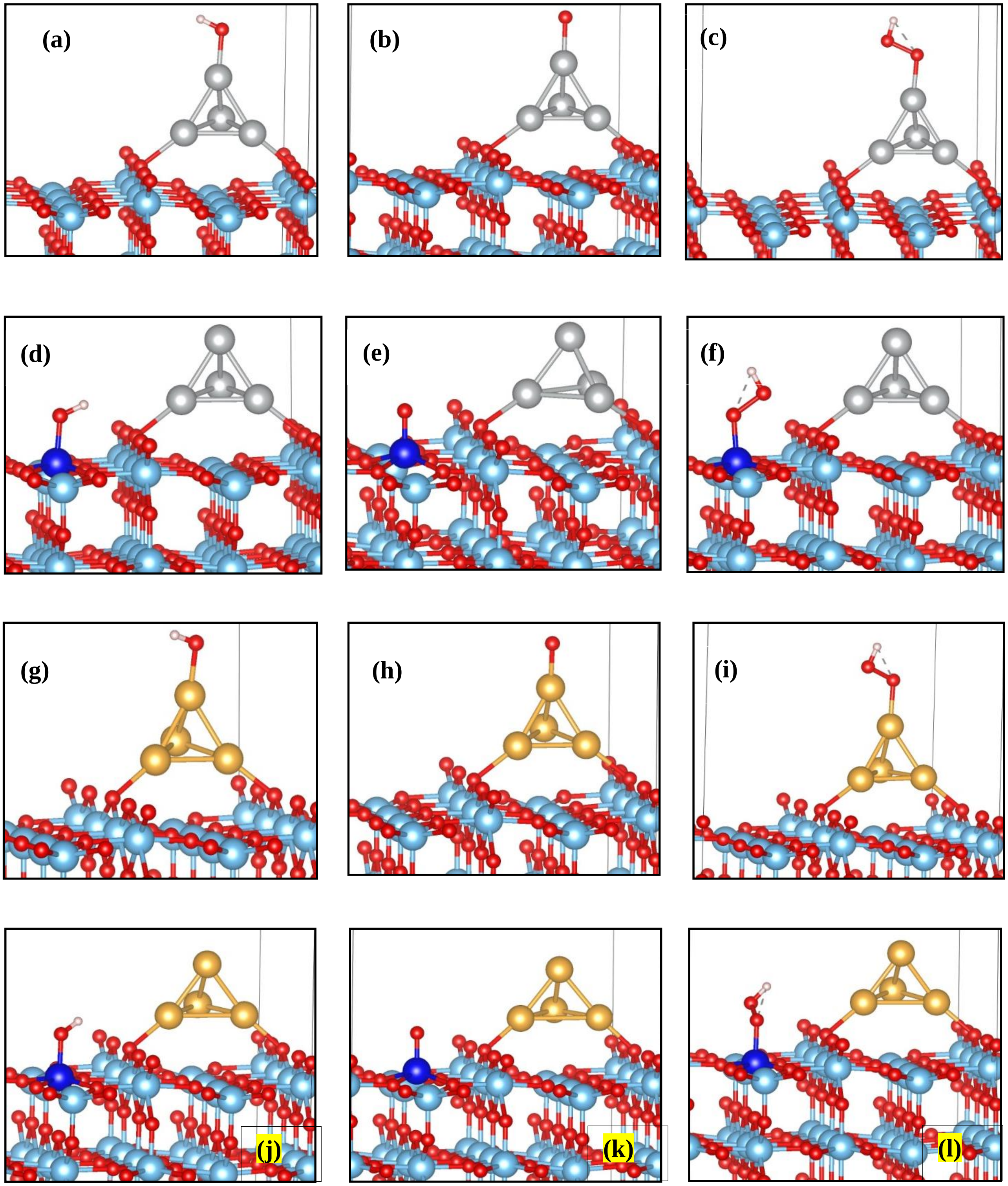

Figure S5: OER optimized intermediates on two bridging oxygen rows Ag_4/TiO_2 , Ag active site (a) OH*-Ag, (b) O*- Ag (c) OOH*-Ag ; Ti active site (d) OH*-Ti, (e) O*- Ti (f) OOH*-Ti ; Au_4/TiO_2 , Au active site (g) OH*-Au, (h) O*- Au (i) OOH*- Au ; Ti active site (j) OH*-Ti, (k) O*- Ti (l) OOH*-Ti.

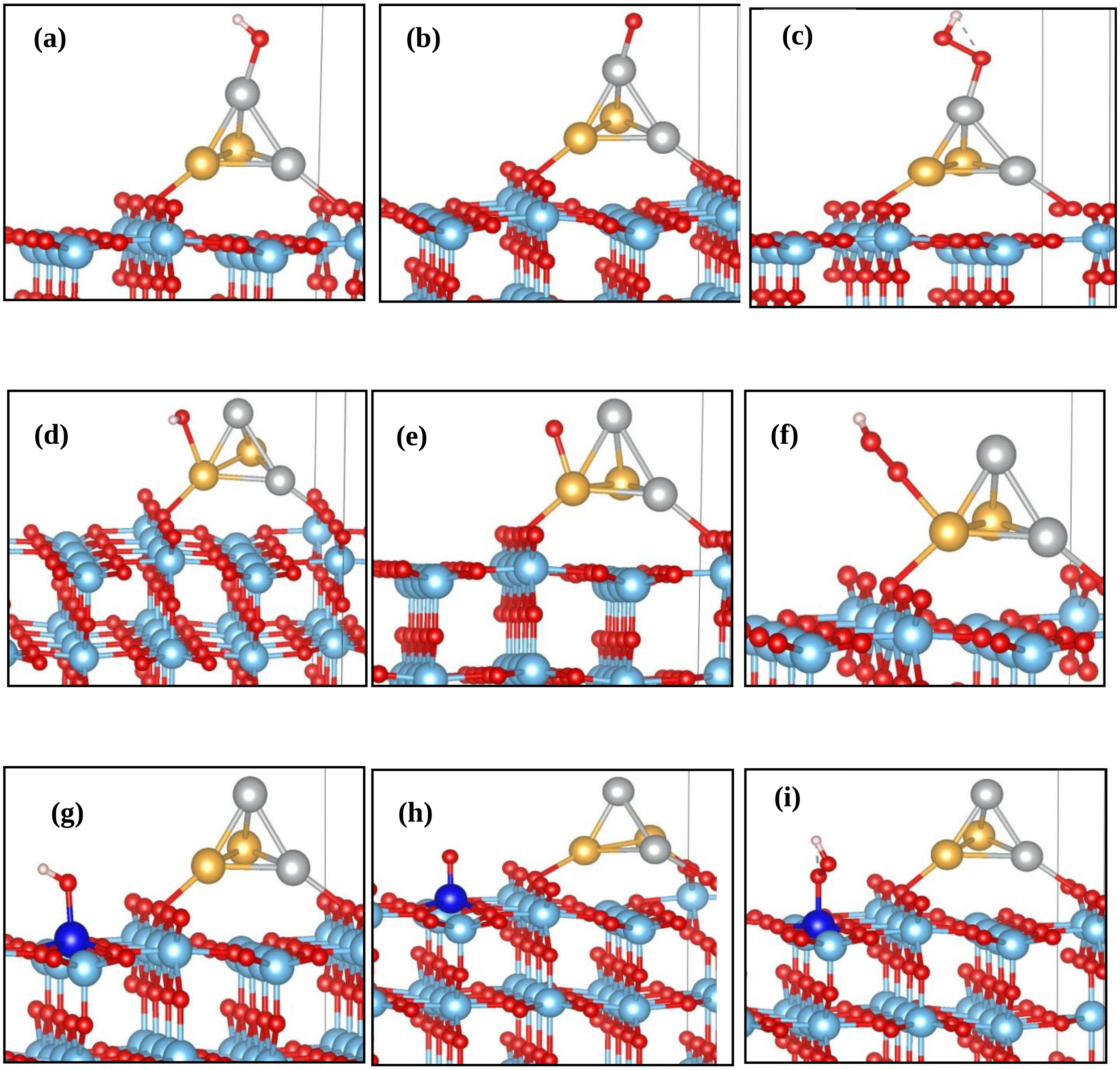

Figure S6: OER optimized intermediates on two bridging oxygen rows Au_2-Ag_2/TiO_2 , Ag active site (a) OH*-Ag, (b) O*- Ag (c) OOH*-Ag ; Au active site (d) OH*-Au, (e) O*- Au (f) OOH*-Au ; Ti active site (g) OH*- Ti, (h) O*- Ti (i) OOH*- Ti.

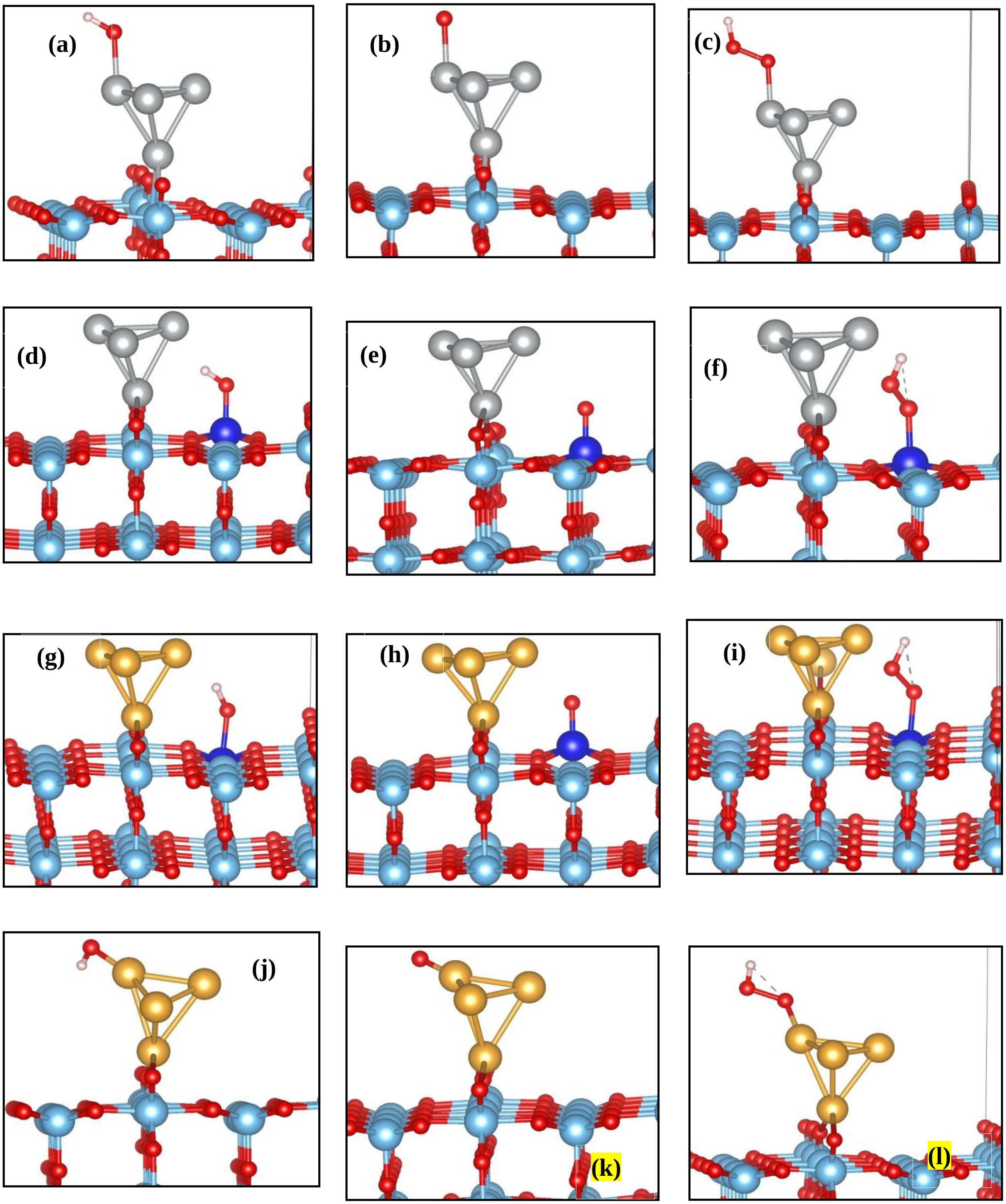

Figure S7: OER optimized intermediates of Ag_4/TiO_2 placed on oxygen vacant site, Ag active site (a) OH*-Ag, (b) O*- Ag (c) OOH*-Ag ; Ti active site (d) OH*-Ti, (e) O*- Ti (f) OOH*-Ti ; Au_4/TiO_2 , Ti active site (g) OH*- Ti, (h) O*- Ti (i) OOH*- Ti ; Au active site (j) OH*-Au, (k) O*- Au (l) OOH*-Au.

3. **Free Energy Diagram:**

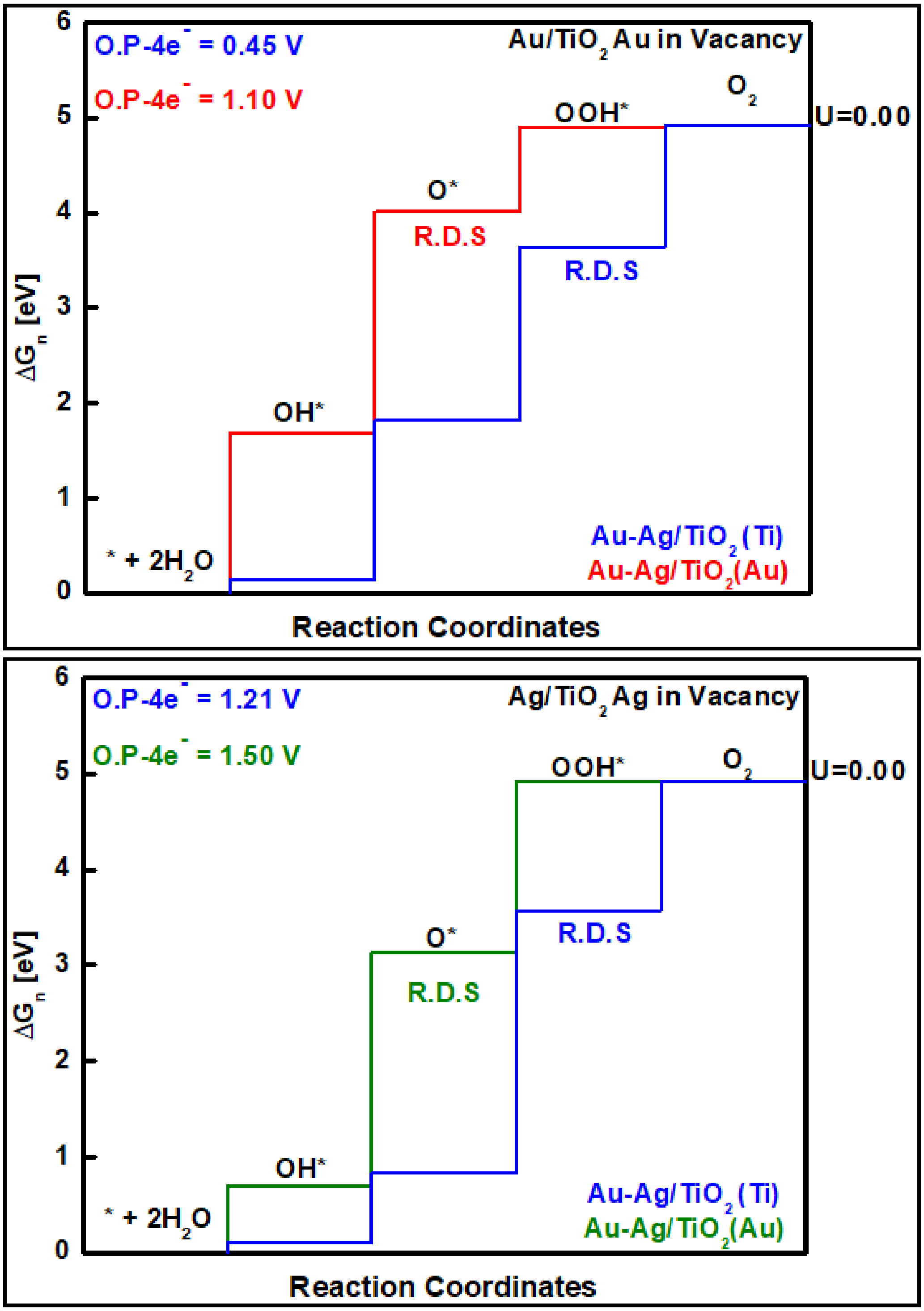

Figure S8: Comparative free energy diagram **(initial model)** for Au/TiO_2 and Ag/TiO_2 on bridging oxygen vacant site.

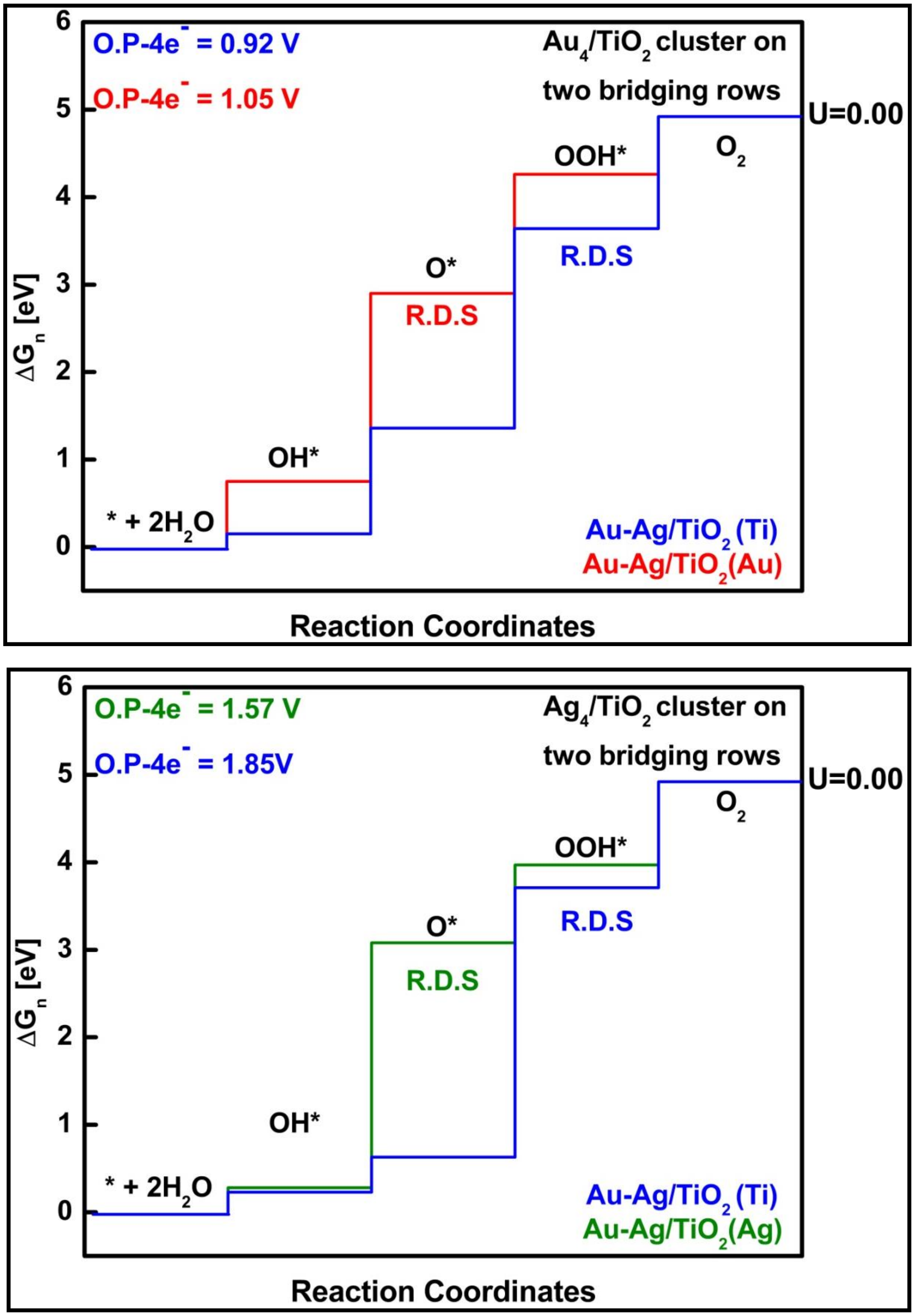

Figure S9: Comparative free energy diagram **(cluster model)** for Au_4/TiO_2 and Ag_4/TiO_2 on two bridging oxygen rows.

4. Band Structure for Au_4/TiO_2 and Ag_4/TiO_2:

Here we have shown the band structures for Au_4/TiO_2 and Ag_4/TiO_2 structures when Au and Ag four atoms are positioned on bridging oxygen vacant position. We found trap states in forbidden gap for both structures. Wang et al., reported band structure calculations on Au_8 atoms cluster on rutile TiO_2 structure. The author placed an Au_8 atom cluster on two bridging rows of TiO_2 surface and found similar trap states in the forbidden gap [4].

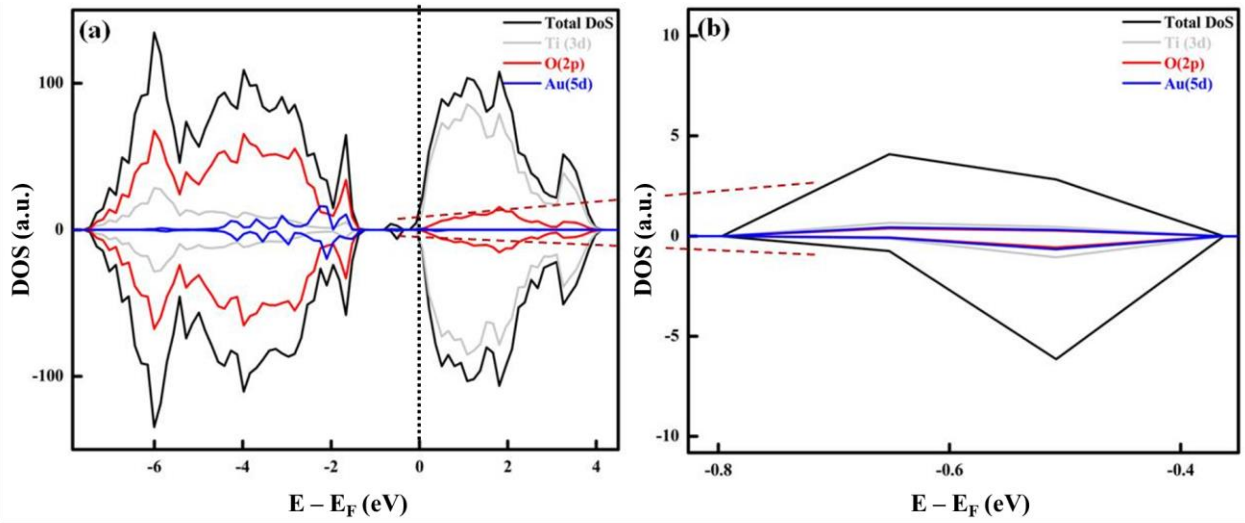

Figure S10: DOS results for four atoms cluster Au_4/TiO_2 on bridging row oxygen vacant site of (a) Enlarged view of trap states which is majorly composed of Au(5d).

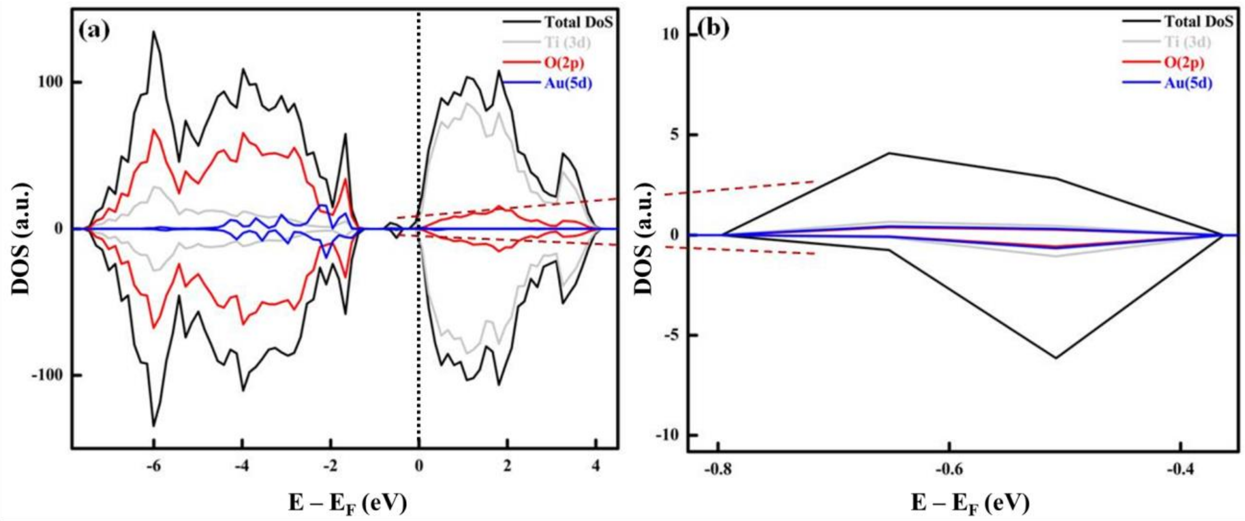

Figure S11: DOS results for four atoms cluster Ag_4/TiO_2 on bridging row oxygen vacant site of (a) Enlarged view of trap states which is majorly composed of Ag(4d).

5. Relative free energies:

In table S2, the relative free energies for initial models shown in figure S3 are given. For the cluster model placed on two bridging rows, shown in figure S5, the relative energies are given in table S3, starting from * + $2H_2O$ (taken as zero energy) for OH* (**ΔG_{OH*}), O* (ΔG_{O*}), O* (ΔG_{OOH*})** and the associated overpotentials for all systems.'*' represents rate-determining steps.

Table S2: Relative free energies for **Initial Model** Ag-TiO_2, replacing bridging O atoms on surface with Ag atom

Table S3: Relative free energies for **'Cluster model'** Ag_4-TiO_2 and Au_4-TiO_2 cluster on two

**System: Ag-TiO_2, replacing bridging O atoms on surface with Ag atom**					
**Intermediates**	**ΔG_{OH*}**	**ΔG_{O*}**	**ΔG_{OOH*}**	**O.P.**	**R.D.S**
Ti active site	0.10	0.84	**3.57***	**1.50 V**	**OOH***
Ag active site	0.70	**3.14***	4.92	**1.21 V**	**O***
System: System: Au-TiO_2, replacing bridging O atoms on surface with Ag atom					
Ti active site	0.14	1.82	**3.64***	**0.45 V**	**OOH***
Au active site	1.68	**4.01***	4.90	**1.10 V**	**O***

bridging oxygen rows

**System: Ag_4-TiO_2, cluster on two bridging oxygen rows**					
**Intermediates**	**ΔG_{OH*}**	**ΔG_{O*}**	**ΔG_{OOH*}**	**O.P.**	**R.D.S**
Ti active site	0.23	0.63	**3.71***	**1.85 V**	**OOH***
Ag active site	0.28	**3.08***	3.97	**1.57 V**	**O***
**System: Au_4-TiO_2, cluster on two bridging oxygen rows**					
Ti active site	0.15	1.36	**3.64***	**1.05 V**	**O***
Au active site	0.75	**2.90***	4.26	**0.92 V**	**OOH***

References:

[1] Nørskov JK, Rossmeisl J, Logadottir A, Lindqvist L, Kitchin JR, Bligaard T, et al. Origin of the overpotential for oxygen reduction at a fuel-cell cathode. The Journal of Physical Chemistry B. 2004;108:17886-92.

[2] Rossmeisl J, Qu ZW, Zhu H, Kroes GJ, Nørskov JK. Journal of Electroanalytical Chemistry2007. p. 83-9.

[3] Valdés Á, Qu ZW, Kroes GJ, Rossmeisl J, Nørskov JK. Oxidation and Photo-Oxidation of Water on TiO2 Surface. The Journal of Physical Chemistry C. 2008;112:9872-9.

[4] Wang S, Gao Y, Miao S, Liu T, Mu L, Li R, et al. Positioning the Water Oxidation Reaction Sites in Plasmonic Photocatalysts. Journal of the American Chemical Society. 2017;139:11771-8.
